# Association of Dose of Inhaled Corticosteroids and Frequency of Adverse Events

**DOI:** 10.1164/rccm.202402-0368OC

**Published:** 2024-08-01

**Authors:** Chloë I. Bloom, Freda Yang, Richard Hubbard, Azeem Majeed, Jadwiga A. Wedzicha

**Affiliations:** ^1^ National Heart and Lung Institute, Imperial College London, London, United Kingdom;; ^2^ Lifespan and Population Health, School of Medicine, University of Nottingham, Nottingham, United Kingdom; and; ^3^ School of Public Health, Imperial College London, London, United Kingdom

**Keywords:** asthma, cardiovascular disease, pulmonary embolism, pneumonia, corticosteroids

## Abstract

**Rationale:**

Inhaled corticosteroids (ICSs) are the cornerstone of asthma treatment and significantly improve morbidity and mortality. Adverse effects of oral corticosteroids are well documented, but less is known about ICS.

**Objectives:**

The aim of this study was to determine the risk of adverse effects from short-term ICS use in people with asthma.

**Methods:**

We conducted observational studies in adults with asthma using two different United Kingdom nationwide datasets: Clinical Practice Research Datalink Aurum and Clinical Practice Research Datalink GOLD. The exposure was incident ICS; the outcomes were a major adverse cardiac event (MACE), arrhythmia, pulmonary embolism (PE), and pneumonia over 12 months. Our main analyses used a cohort method with stabilized inverse probability treatment weighting to balance confounding between exposed and unexposed patients. Secondary analyses included nested case–control studies and self-controlled case series. ICS use was treated as both a categorical and a continuous variable. Absolute risk was estimated using weighted flexible parametric models.

**Measurements and Main Results:**

Among 162,202 patients in our main cohort, there was an association with all outcomes at the medium daily ICS dose or higher (hazard ratios [HRs] at 201–599 μg: MACE, 2.63 [95% confidence interval (CI), 1.66–4.15]; arrhythmia, 2.21 [95% CI, 1.60–3.04]; PE, 2.10 [95% CI, 1.37–3.22]; and pneumonia, 2.25 [95% CI, 1.77–2.85]; HRs at ≥600 μg: MACE, 4.63 [95% CI, 2.62–8.17]; arrhythmia, 2.91 [95% CI, 1.72–4.91]; PE, 3.32 [95% CI, 1.69–6.50]; and pneumonia, 4.09 [95% CI, 2.98–5.60]). There were no associations with lower doses of ICSs. Secondary analyses produced similar results. The number needed to harm using 12 months of ICS at 201 to 599 μg was as follows: MACE, 473 (95% CI, 344–754); arrhythmia, 567 (95% CI, 395–1,006); PE, 1,221 (95% CI, 744–3,388); and pneumonia, 230 (95% CI, 177–327). The number needed to harm using ICS at ≥600 μg was as follows: MACE, 224 (95% CI, 148–461); arrhythmia, 396 (95% CI, 228–1,523); PE, 577 (95% CI, 309–4,311); and pneumonia, 93 (95% CI, 69–141).

**Conclusions:**

Short-term use of low-dose ICS was not associated with adverse effects. Moderate to high daily ICS doses were associated with an increased risk, but low frequencies, of cardiovascular events, PE, and pneumonia. It is important for clinicians to adhere to guideline recommendations to use the lowest effective ICS dose.

**At a Glance Commentary**

**Scientific Knowledge on the Subject**

Inhaled corticosteroids (ICSs) are fundamental for the treatment of patients with asthma. Adverse effects from oral corticosteroids are well recognized, including risk of cardiovascular disease.

**What This Study Adds to the Field**

By applying a triangulation methodological approach to two different large observational datasets, we found significant risk of four adverse outcomes (cardiovascular and pulmonary) associated with using medium- and high-dose ICSs, each with a low frequency of events. There was no risk of adverse outcomes associated with 12-month use of low-dose ICS.

Inhaled corticosteroids (ICSs) are the foundation of asthma treatment. They significantly improve patients’ quality of life, reduce respiratory symptoms, improve lung function, prevent asthma exacerbations, and reduce mortality (1). Guidelines recommend prescribing the lowest possible dose that achieves good asthma control (2, 3). Remarkably, 80–90% of the maximal benefit possible from ICSs is achieved from low-dose ICS alone (100–200 μg fluticasone equivalent) (4, 5). Yet in clinical practice, ICSs are frequently prescribed at inappropriately high doses in patients with mild to moderate asthma that could be well controlled with lower doses (6).

The cumulative adverse effects of oral corticosteroids in asthma are well documented and include cardiovascular disease (CVD), osteoporosis, cataracts, adrenal suppression, obesity, and diabetes (7). Even short-term use of oral corticosteroids, in those with or without asthma, can have serious adverse effects (8). Although there are far fewer available data regarding the safety profile of ICSs, several studies have demonstrated significant systemic absorption, including two recent studies. First, on the basis of hypothalamic–pituitary axis data obtained from multiple randomized clinical trials, approximately two-thirds of high-dose ICSs appeared to be systemically absorbed (9). Second, a large-scale metabolomic profiling study of plasma revealed extensive adrenal suppression associated with low-dose ICS use in asthma (10).

Several studies have investigated potential adverse effects of ICS use in chronic obstructive pulmonary disease (COPD), focusing particularly on pneumonia, as well as bone density and diabetes (8). However, there is a lack of studies in asthma, with preceding studies reporting conflicting findings (11).

In this study, we aimed to determine the risk of adverse effects from short-term ICS use in people with asthma. We used two large different nationwide cohorts of asthma patients naive to ICSs at the start of follow-up and applied three different study designs; this triangulation approach was taken to improve the robustness of findings.

## Methods

### Data Sources

Clinical Practice Research Datalink (CPRD) Aurum is a nationwide database of routinely collected information, from electronic medical records in general practitioner (GP) practices using EMIS software (EMIS Health), covering approximately 20% of the population of the United Kingdom (https://cprd.com). CPRD GOLD is a second nationwide database, from GP practices using Vision software (Cegedim Healthcare Solutions), covering approximately 5% of the United Kingdom. The Aurum database was used for all the studies. To validate findings, the GOLD database was also used when sufficient sample size was available. Patients’ records were individually linked to Hospital Episode Statistics (English hospital admission data) and Office for National Statistics mortality data.

This study is based in part on data from the Clinical Practice Research Datalink obtained under license from the UK Medicines and Healthcare products Regulatory Agency. The data is provided by patients and collected by the NHS as part of their care and support. The interpretation and conclusions contained in this study are those of the authors alone. Hospital Episode Statistics (HES) and Office for National Statistics (ONS) data: Copyright © (2020), re-used with the permission of The Health and Social Care Information Centre. All rights reserved. The protocol for this research was approved by CPRD’s Research Data Governance Process (protocol number: 20_090).

### Study Population and Designs

Patients with asthma were eligible if they were ≥18 years of age, were not yet started on ICSs at study entry (at least by 1 day), contributed data between January 1, 2004, and 2019, and had at least 1 year of data before study entry. This cohort was termed the “ICS new-user cohort.” Follow-up ended at the earliest of January 1, 2019, last data collection, date or death. From the ICS new-user cohort, additional populations were drawn for the secondary analyses: nested case–control studies and a self-controlled case series (SCCS; *see* Figure E1 in the data supplement). Asthma was defined as the presence of at least two asthma CPRD Read codes within two years of study entry (12) (*see* Table E1).

### Exposure

From the new-user cohort, patients were defined as “exposed” if ICSs were prescribed within the first year of cohort entry. ICS was categorized in several different ways (beclomethasone equivalent): *1*) by the dose of the first ICS prescription: 400 μg (low), 401–1,000 μg (medium), or ≥1,000 μg (high) as per national guideline recommendations (British Thoracic Society and Scottish Intercollegiate Guidelines Network 2019 guideline for the management of asthma, Table 12, and Global Initiative for Asthma 2023, Boxes 3–14) (2, 3); *2*) by calculated average daily dose during the study period as a continuous variable; *3*) by categorization of calculated average daily dose (≤200 μg [low], 201–599 μg [medium], or ≥600 μg [high], reflecting real-world adherence); and *4*) by type of ICS (beclomethasone, budesonide, or fluticasone).

So that the exposed and unexposed groups were as similar as possible according to asthma severity, unexposed patients were excluded from the cohort if they did not commence ICSs within 2 years.

We also extracted data on citalopram use, to act as a negative control in our study, to provide insights into possible bias.

### Outcomes

The four primary outcomes were identified in Hospital Episode Statistics using International Classification of Diseases, 10th Revision, codes: *1*) major adverse cardiac event (MACEs): myocardial infarction (codes I21–I23), cerebrovascular accident (codes I63 and I69), or cardiovascular death (Office for National Statistics data); *2*) arrhythmia (codes I41 and I47–I49); *3*) hospitalized community-acquired pneumonia (code J18); and 4) pulmonary embolism (PE; code I126). In the secondary analyses, because of the small number of events, a composite variable, CVD, was used and included MACEs and arrhythmia.

The negative control outcomes included hospital admission for urinary tract infection (code N39), forearm fracture (code S52), abdominal pain (including diverticular disease; codes R10 and K57), and cellulitis (code L03). These were chosen as they are relatively common reasons for hospital admission in the study population but not known to be associated with ICS use; any association found would suggest residual confounding in the model.

### Potential Confounders and Modifiers

The following variables were considered *a priori* to be potential confounders (*see* the directed acyclic graphs in Figure E2): sex, age, GP practice, calendar year of cohort entry, body mass index, smoking history (current/former/never), socioeconomic status (Index of Multiple Deprivation), history of CVD (including hypertension, ischemic heart disease, and arrhythmias), previous hospital admissions (pneumonia and CVD), type 2 diabetes, cerebrovascular accident, COPD, chronic renal failure, anxiety, depression, cancer, oral contraceptives, hormone replacement therapy, bronchodilators (short-acting β-agonists [SABAs] and long-acting β-agonists), and GP consultations in the year before study entry. Variables with missing values (body mass index and smoking) were modeled with an unknown category. Patients prescribed oral corticosteroids in the year before or any time during follow-up were excluded from the main analysis and the SCCS population but not from the case–control analysis (allowing the effect of oral corticosteroids to be observed).

### Statistical Analysis

To avoid the potential limitation of confounding by indication, we undertook several measures. In the main analysis, we compared a cohort of patients with asthma (mostly mild asthma) newly initiated on ICSs against patients with asthma (mostly mild asthma) just before initiating ICSs and applied propensity score methodology (a common approach to minimize confounding by indication) including several markers of asthma severity and control. We next conducted several secondary analyses, each intended to minimize confounding using different approaches, and we undertook numerous analyses to test for residual confounding, as well as using two completely different patient databases. Using a triangulation approach, applying several methods each with different biases, increases the reliability and robustness of estimates (13).

Of three different study designs applied, the cohort was used in the main analysis, and the nested case–control studies and SCCS were used for the secondary analyses.

#### Main analysis

Patients were eligible if they had two or more consecutive ICS prescriptions. Patients were censored at the earliest of 12-month follow-up, change in exposure status (if an ICS user stopped using ICS or a nonuser commenced ICS), first outcome event, or cohort end.

To achieve exchangeability (symmetry in the probability distribution), conditional on the available covariates, between the exposed and unexposed groups, stabilized inverse probability treatment weighting (IPTW) was used. Weights were estimated using probability of exposure assignment derived from multivariable logistic regression models accounting for confounders. Absolute standardized differences were calculated to assess covariate balance, with differences <0.10 indicating good balance. Weighted Cox proportional-hazard models were fit to estimate the relative risk. A restricted cubic spline was applied to estimate the risk associated with the average daily ICS dose.

To determine the absolute risk difference, weighted flexible parametric models were fitted, from which the number needed to harm was calculated.

Models were stratified by factors considered *a priori* to potentially modify the association. The variables included age (cutoff, 50 years), blood eosinophil count (cutoff, 0.3 × 10^9^/L), and comorbid CVD.

As a sensitivity analysis, to determine if acute respiratory infection was a mediator for MACEs, all patients with respiratory infections during follow-up or at the time of the MACE were excluded.

In addition to the negative control analyses, we also assessed for evidence of residual confounding by conducting an e-value analysis (where a large e value implies that considerable unmeasured confounding would be needed to explain away the effect estimate).

To determine if ICS drug type affected the risk, two active-comparator, new-user IPTW models were conducted as a subanalysis: beclomethasone compared with fluticasone and beclomethasone compared with budesonide. The drug type was based on the first inhaler prescribed. The weights estimated included the same confounders as the main models but also included ICS dose.

#### Secondary analyses

To strengthen confidence in our findings, *1*) we repeated the analysis in a second (smaller) database, CPRD GOLD, and *2*) we conducted two other study designs that have different advantages and limitations from our main analysis.

1. IPTW cohort (CPRD GOLD dataset): The same methods were applied as described above for the Aurum IPTW cohort. Because of the smaller sample size, there was insufficient power to assess PE.
2. Nested case–control (CPRD Aurum dataset): Cases and control subjects were nested from the ICS new-user cohort, matching three or four control subjects to each case by year of birth, sex, and GP practice, using incidence density sampling. Conditional logistic regression was applied to estimate the relative odds. Confounders listed above were included in the models. The exposure window was divided into four time periods: 0–30, 31–90, 91–180, and 180–365 days; each window represents the period during which the last ICS prescription occurred.
3. Nested case–control (CPRD GOLD dataset): An identical case–control design was used as above, except that the patients were obtained from an ICS new-user cohort drawn from CPRD GOLD. Because of the much smaller GOLD dataset, there was insufficient power to estimate the risk of PE or the association between citalopram and pneumonia.
4. SCCS: In this design, each patient acts as their own control, therefore implicitly controlling for time-invariant confounding (e.g., genetics, comorbidities) (14). Incidence rate ratios (IRRs) were calculated using fixed-effects conditional Poisson regression adjusted for age, comparing the rate of events during exposed periods with that during all other observed periods. This allowed event rates to be estimated before, during, and after ceasing ICSs.

To ensure valid and unbiased estimates, certain assumptions must be met (14, 15). First, the outcome should be independent of previous outcomes. Therefore, only incident outcome events were included. The second assumption is that the outcome does not affect the probability of exposure. If the outcome affects the risk of exposure only within a short time frame, this potential bias can be removed by using a preexposure period, which is excluded from the analysis. To be included in the SCCS, each patient must have had the exposure and outcome during the observation period, which was set at 12 months before exposure and up to 12 months after. Only CVD could be assessed, as pneumonia violated the assumptions, and there were too few patients to assess PE.

#### All analyses and models

The initial 7 days of event data after the first ICS prescription were excluded to remove potential protopathic bias (that ICSs were prescribed because of symptoms of the outcome).

Thirty days were added to the last ICS prescription, assuming the patient continued to use that inhaler for about 1 month.

All analyses were conducted using Stata version 18 (StataCorp LLC).

## Results

### Characteristics of the ICS New-User Cohort

In total, 162,202 patients were eligible for the ICS new-user cohort (*see* Table E1 and Figure E3). Before weighting, patients in the ICS group compared with the non-ICS group were marginally older and obese, a slightly higher proportion had comorbidities, and there was higher use of SABAs. After weighting, the ICS and non-ICS groups were well balanced.

The proportion of exposed patients by dose of first ever ICS prescription was as follows: low dose, *n* = 62,778 (65.1%); medium dose, *n* = 28,832 (29.9%), and high dose, *n* = 4,831 (5.0%). Categorizing by average daily dose, 59,909 patients (62.1%) were taking ≤200 μg, 31,054 (32.2%) were taking 201–599 μg, and 5,478 (5.7%) were taking ≥600 μg. At baseline, 13.9% of patients were started on combination inhalers with both ICSs and long-acting β-agonists, instead of ICSs alone, and <1% were also prescribed leukotriene receptor antagonists.

### Incidence Rates in the ICS New-User Cohort

The incidence of each adverse outcome was low but was highest for hospitalized pneumonia (incidence rate per 1,000 person-years: MACE, 1.5 [95% confidence interval (CI), 1.32–1.71]; arrhythmia, 1.64 [95% CI, 1.45–1.86]; PE, 0.84 [95% CI, 0.74–0.98]; and pneumonia, 3.40 [95% CI, 3.18–3.65]). The crude incidence rate was marginally higher in the ICS group for all outcomes and increased with higher ICS dose (*see* Table E2).

### Association between ICS and Adverse Outcomes in the Main Analysis

The Kaplan-Meier plots for each outcome show increasing risk in ICS users over time (*see* Figure E4).

**Figure 1.**
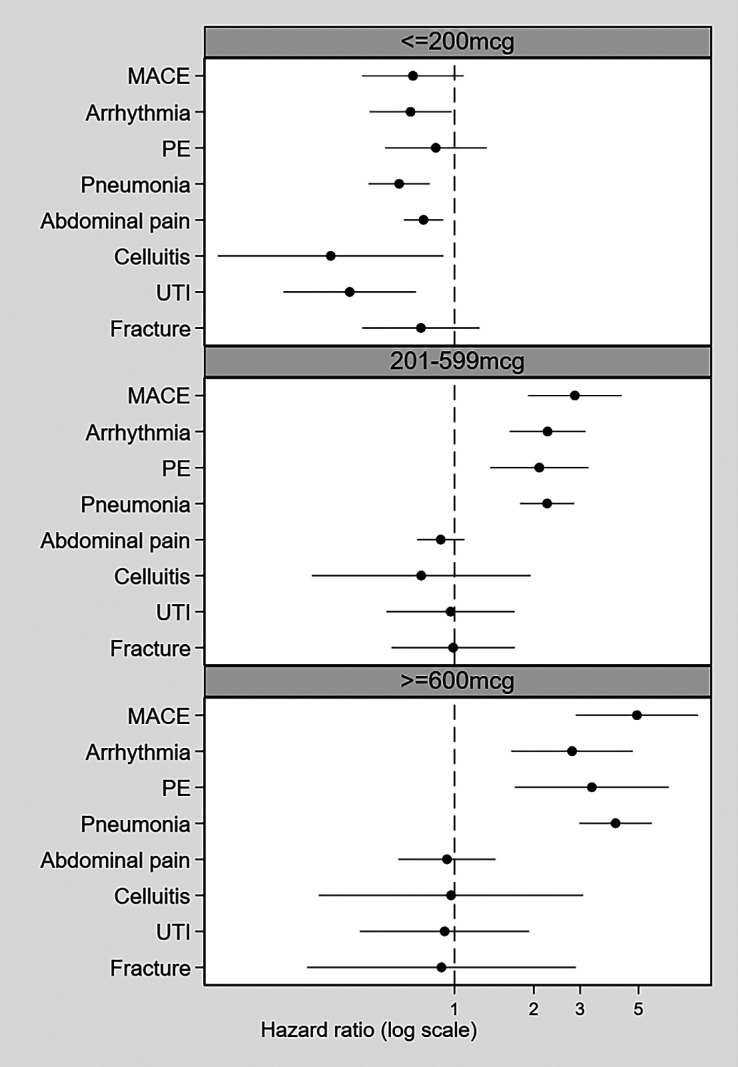
Association between ICS use and outcomes, categorized by average daily ICS dose (main analysis: Clinical Practice Research Datalink Aurum inverse probability treatment weighting cohort). ICS = inhaled corticosteroid; MACE = major adverse cardiovascular events; PE = pulmonary embolism; UTI = urinary tract infection.

As a categorical variable, ICS use was significantly associated with all adverse outcomes only if the average daily dose was greater than 200 μg (*see* Table E3; Figure 1) or when the first dose prescribed was at least a medium dose (*see* Figure E5).

**Figure 2.**
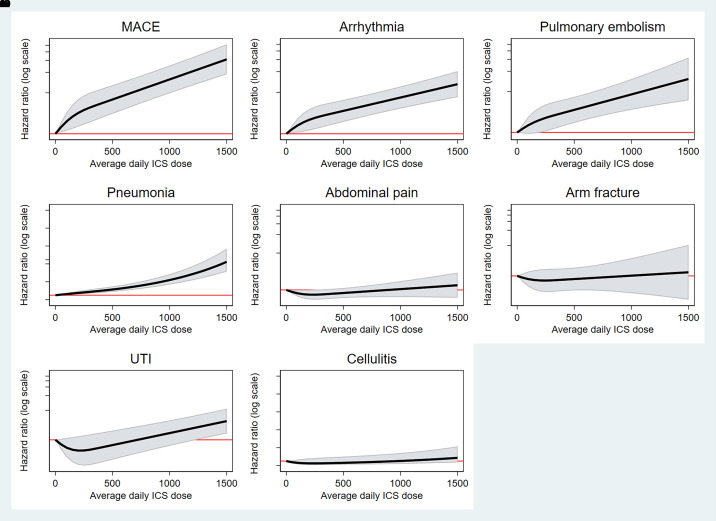
Association between average daily ICS dose as a continuous outcome and outcomes (main analysis: Clinical Practice Research Datalink Aurum inverse probability treatment weighting cohort). ICS = inhaled corticosteroid; MACE = major adverse cardiovascular events; UTI = urinary tract infection.

Considering ICS as a continuous variable, the risk of each adverse outcome increased significantly with increasing daily ICS dose (Figure 2).

In the negative control outcome analyses, no association was observed between ICS and hospitalization for urinary tract infection, cellulitis, abdominal pain, or forearm fracture (Figures 1, 2, and E5). From the e-value analysis, the observed hazard ratios could be explained away only by an unmeasured confounder with a very high hazard ratio, above and beyond the measured confounders (*see* Table E4).

In the sensitivity analysis, there was negligible difference to the effect estimate for MACEs (201–599 μg: hazard ratio, 2.61 [95% CI, 1.60–4.26]; ≥600 μg: hazard ratio, 5.07 [95% CI, 2.52–10.22]).

### Association between ICS and Adverse Outcomes in the Secondary Analyses

#### IPTW cohort in the CPRD GOLD dataset

There was a significant association with dose–response relationships between ICS and CVD and ICS and pneumonia (*see* Figure E6 and Table E5).

#### Nested case–control in the CPRD Aurum dataset

**Figure 3.**
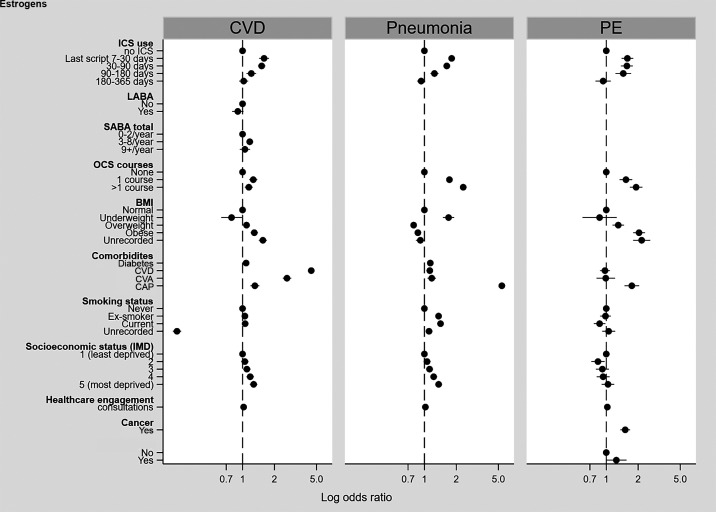
Association between inhaled corticosteroid (ICS) use and outcomes by time since last ICS prescription (secondary analysis: Clinical Practice Research Datalink Aurum nested case–control). BMI = body mass index; cap = community-acquired pneumonia; cva = cerebrovascular accident; CVD = cardiovascular disease; IMD = Index of Multiple Deprivation; LABA = long-acting β-agonist; OCS = oral corticosteroid; PE = pulmonary embolism; SABA = short-acting β-agonist.

There were significantly increased odds of CVD, PE, and pneumonia at least 90 days after the last ICS prescription (Figure 3; *see* Tables E6–E8). There was no association with the negative control exposure, citalopram (*see* Figure E7).

#### Nested case–control in the CPRD GOLD dataset

There were significantly increased odds of CVD and pneumonia at least 30 days after the last ICS prescription (*see* Figures E8 and E9 and Tables E9 and E10). There was no association with CVD and the negative control exposure, citalopram (*see* Figure E8).

#### SCCS

**Figure 4.**
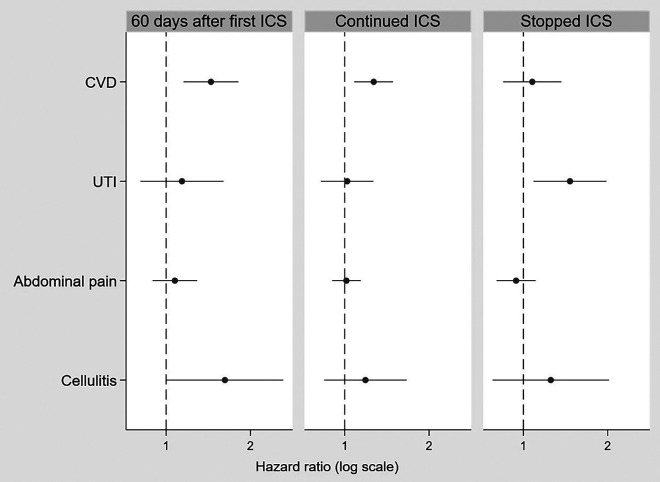
Association between ICS use (during exposure and after exposure stopped) and outcomes: CVD and the negative controls of UTI, abdominal pain, and cellulitis (secondary analysis: Clinical Practice Research Datalink Aurum self-controlled case series). CVD = cardiovascular disease; ICS = inhaled corticosteroid; UTI = urinary tract infection.

CVD risk increased significantly within 60 days after ICS exposure (IRR, 1.45 [95% CI, 1.15–1.81]; *P* < 0.01) and continued during ICS exposure (IRR, 1.27 [95% CI, 1.08–1.49]; *P* < 0.01) but reduced to null with no significant association after ICS exposure stopped (IRR, 1.08 [95% CI, 0.85–1.37]; *P* = 0.55) (Figure 4; *see* Table E11). No increased risk was observed for the negative control outcomes of urinary tract infection, cellulitis, and abdominal pain.

### Absolute Risk (Main Analysis)

**Figure 5.**
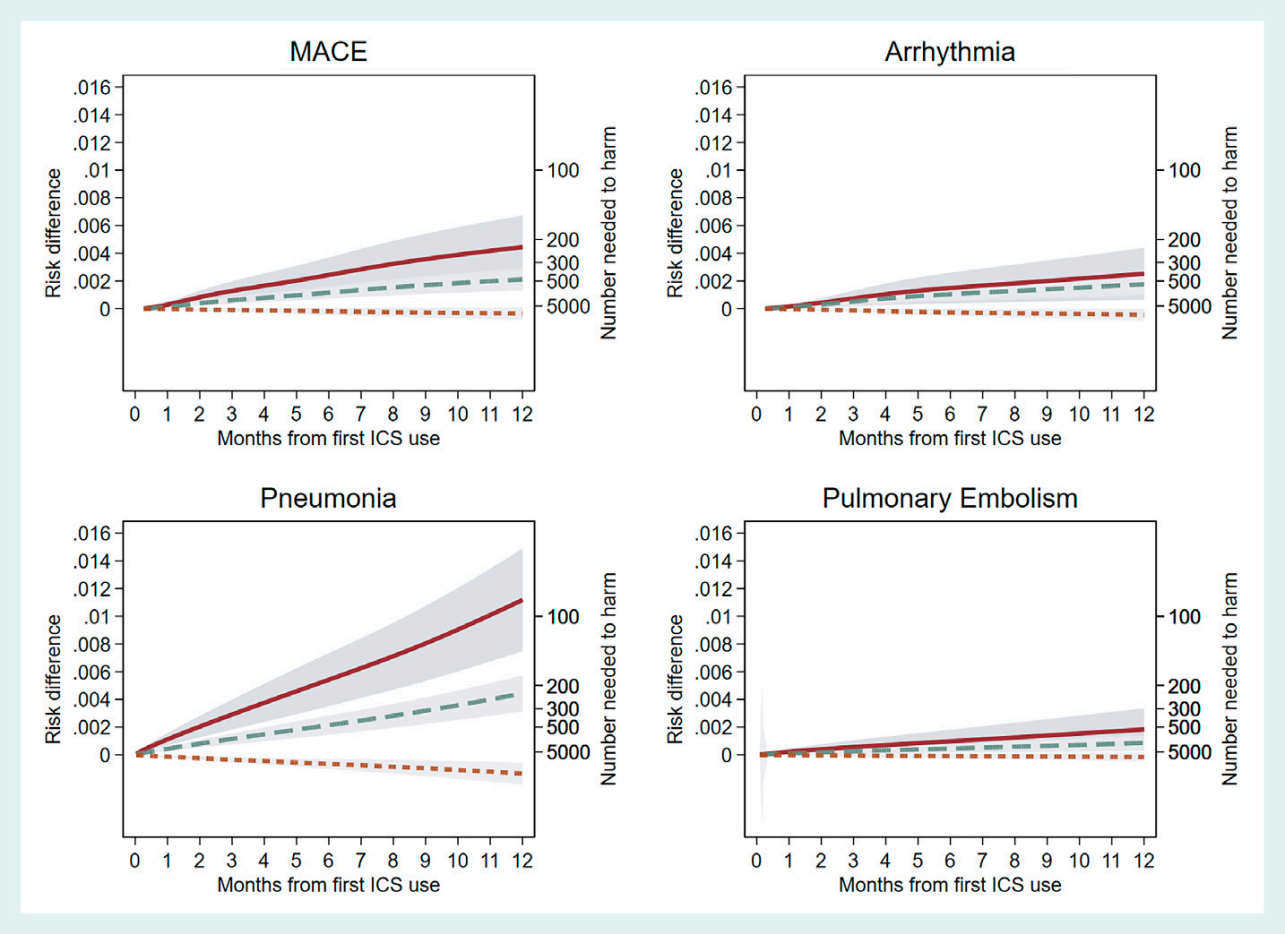
Absolute risk of each outcome associated with categorized average daily dose of inhaled corticosteroid (ICS) (main analysis: Clinical Practice Research Datalink Aurum inverse probability treatment weighting cohort). Red line denotes high-dose ICS, dashed green line denotes medium-dose ICS, and red dotted line denotes low-dose ICS. Gray shading denotes the 95% confidence interval around the estimate. MACE = major adverse cardiovascular events.

The weighted risk difference varied for each outcome. The highest absolute risk was observed for pneumonia and high-dose ICS use. There was no significant risk for any outcome with 12 months of low-dose ICS (Figure 5). The numbers needed to harm for 3 months and 12 months of ICS use are shown in Table 1.

**Table 1.** Absolute Risk of Each Adverse Outcome, Categorized by Average Daily Dose of Inhaled Corticosteroid and Reported for 3 and 12 Months of Inhaled Corticosteroid Use (Main Analysis: Clinical Practice Research Datalink Aurum Inverse Probability Treatment Weighting Cohort)

Average Daily ICS Dose	Adverse Outcome	3-mo Use		12-mo Use	
		NNH	95% CI	NNH	95% CI
201–599 μg	MACE	1,515	899–4,807	473	344–754
	Arrhythmia	CI includes 0		567	395–1,006
	PE	CI includes 0		1,221	744–3,388
	Pneumonia	3,390	1,776–36,954	230	177–327
≥600 μg	MACEs	731	398–4,403	224	148–461
	Arrhythmia	2,499	1,281–50,684	396	228–1,523
	PE	1,031	537–12,789	577	309–4,311
	Pneumonia	718	407–2,997	93	69–141

*Definition of abbreviations*: CI = confidence interval; ICS = inhaled corticosteroid; mo = months; MACE = major adverse cardiovascular event; NNH = number needed to harm; PE = pulmonary embolism.

The lowest average daily ICS dose (≤200 μg) is not shown, as there was no risk (the CIs included zero) for all outcomes.

### Stratified Analyses (Main Analysis)

There was no modification of the association by age or blood eosinophil count, but there was significant modification by CVD comorbidity for MACEs: patients without comorbid CVD were at significantly greater risk than those with comorbid CVD (*P* < 0.05, Wald test; *see* Figure E10).

### Comparing Different ICS Drugs

The differences in the relative risk of each outcome between fluticasone (*n* = 8,537) and beclomethasone (*n* = 79,104) and between budesonide (*n* = 8,525) and beclomethasone (*n* = 79,104) were not significant (*see* Figure E11 and Table E12). Although the risk of pneumonia was higher with fluticasone compared with beclomethasone, this did not reach significance, which may reflect insufficient power in this subanalysis.

## Discussion

In this large observational study from two different clinical databases, we found increased risk of CVD (MACEs and arrhythmia), PE, and pneumonia associated with short-term ICS use. We observed a dose-dependent relationship whereby only patients using at least medium-dose ICS had an elevated risk. The relative risk of each outcome was significantly raised, about two- to fivefold. However, at an individual level, the absolute risk of each adverse outcome was low over the 12-month follow-up period. Furthermore, the risk increased over time, such that the risk was not significant for arrhythmia or PE with only 3 months’ use of medium-dose ICS. Critically, the risk of an asthma exacerbation for patients not managed with ICS, even for mild asthma, is much greater than the risk of any of the adverse events we investigated. For example, in the Novel START trial (an open-label trial to reflect real-world practice), the rate of exacerbations in those not using ICS was 0.40 exacerbations per patient per year (16), more than 20 times higher than the highest adverse outcome rate we observed (0.02 cases of pneumonia per patient per year, using high-dose ICS). Our findings therefore support current guideline recommendations indicating that to avoid unnecessary corticosteroid exposure, through either oral corticosteroid use to treat an exacerbation or excess maintenance ICS doses, an ICS should be prescribed at the lowest dose effective to control a person’s asthma (2, 3). In addition, our findings add weight to the concept that corticosteroid stewardship should include high-dose ICS (17) and emphasize the benefits of seeking to step down patients from high-dose ICS when possible, including biologic-treated patients with severe asthma (18). Asthma reviews and structured medication reviews, both part of the National Health Service GP contract in England, in primary care should be used as key opportunities to review ICS use in people with asthma to ensure that they are prescribed at the lowest effective dose.

Previous studies have observed that oral corticosteroids, regardless of the underlying condition they are treating, increase the risk of developing CVD. Therefore, if even low-dose ICSs lead to systemic absorption (9, 10), we would expect that ICS can also cause CVD. Several studies have suggested that asthma is an independent risk factor for coronary heart disease, including an association with carotid plaque formation, but these studies did not sufficiently account for asthma medication (including corticosteroids) (19). We recently showed comprehensively that asthma itself does not cause coronary heart disease, indicating that associations previously described may have been mediated directly through asthma medication (20). Furthermore, these studies found that more severe or persistent asthma was associated with a higher risk of CVD, yet their definition of a patient with more severe or persistent asthma was one with higher corticosteroid use. Corticosteroids activate glucocorticoid receptors, which are expressed throughout the body; excessive activation leads to insulin resistance, hypertension, and dyslipidemia. Corticosteroids also directly exert endothelial effects, with some experimental studies demonstrating glucocorticoid-induced impaired endothelial function (21). Our understanding is limited by the lack of studies on a complex interaction that must consider multiple factors, including the activation of glucocorticoid and mineralocorticoid receptors, the timing of corticosteroid exposure, and the condition of the endothelium due to underlying pathology.

We postulated that acute respiratory infection secondary to ICS use may have precipitated MACEs (15). However, the removal of patients with respiratory infections during follow-up, or at the time of MACEs, did not mitigate this risk. One factor did appear to modify risk: people with no recorded diagnoses of comorbid CVD were at greater risk than those with recorded diagnoses of CVD. This could be due to a lack of secondary prevention in those with undiagnosed, underlying CVD.

Our finding that ICS use was associated with arrhythmia parallels findings from studies investigating corticosteroid-related arrhythmia (22, 23). Asthma studies have shown an increased risk of arrhythmia in patients using ICS compared with those not using ICS (24). Although the underlying mechanism is unknown, in animal models, high doses of corticosteroids affect myocardial contractility and peripheral vascular resistance (25).

An increased risk of PE has been reported in small observational studies, including people with asthma and other steroid-treated conditions, as well as in people with Cushing’s syndrome (26–28). In healthy volunteers, short bursts of high-dose corticosteroids induce a procoagulant state (29, 30). Moreover, in an experimental asthma study, a procoagulant environment was found in severe asthma (using high-dose ICSs), and medium-dose ICSs were associated with suppression of fibrinolytic factors (31).

ICS is known to be an independent risk factor for pneumonia in patients with COPD (32, 33). Postulated biological mechanisms include modulation of the innate and adaptive immune system, increasing bacterial load and changing the microbial composition in the airways. It is therefore highly plausible that ICSs confer a similar risk in asthma. There has been a small number of asthma observational studies; each revealed an increased risk, but the amount of evidence they provided was limited by their moderate risk of bias (11). A meta-analysis of 26 international asthma ICS trials reported that the risk of pneumonia, on the basis of reporting of “pneumonia” adverse events, actually decreased with ICS use (34). However, as the authors noted, adverse events were likely misclassified (many may have been asthma exacerbations, as radiology was not required to make the diagnosis of pneumonia); instead, the focus should be on serious adverse events, which were likely to be correctly diagnosed and involved a hospital visit and radiological diagnosis. Notably, although the total number of serious adverse pneumonias was low, there were more events in people using ICSs (2.9 per 1,000 person-years) than in those using placebo (2.1 per 1,000 person-years). In a meta-analysis of trials comparing medium- versus high-dose ICS, although not powered to detect such small differences, there were double the number of serious adverse pneumonia events among the high-dose triple-therapy ICS users (11 events in 2,004 patients) as the medium-dose triple-therapy ICS users (5 events in 1,999 patients) (35). We also found that, although not statistically significant, perhaps because of the much smaller sample size, fluticasone appeared to have the strongest association with hospitalized pneumonia, as reported in earlier studies (11).

### Strengths and Limitations

This research question cannot be answered using a randomized controlled trial, as the sample size required would need to be enormous, particularly if also wishing to assess for a dose–response effect. Strengths of this observational study include the size of the study, the use of nationally representative data, leverage of two different datasets of patients, the use of a new-user study design to ensure a clear temporal sequence, the application of three different study designs (consistency in findings across designs with different strengths and weaknesses improves overall confidence), and the use of multiple negative control studies to detect evidence of residual confounding. We are not aware of any study that has applied such a robust approach.

There were also limitations. The first seven days of data after ICS prescription were removed in all models to remove the risk of protopathic bias (that ICSs were prescribed because of symptoms of the outcome), but this also lowered the effect estimate toward the null. There is a risk of misdiagnosis and therefore misclassification of asthma, but this should be a systematic error rather than bias, as both exposed and unexposed were diagnosed and treated with ICSs; therefore, this potential error would move the estimate toward the null. Furthermore, patients with high eosinophil counts are less likely to be misclassified, and the stratified analysis revealed increased outcome risk associated with both low and high eosinophil counts. Even with the exhaustive methodological approaches that we applied, it is still possible that an increase in asthma severity in this population of predominantly patients with mild asthma could have increased their risk of hospitalization for MACEs, arrhythmias, PE, and pneumonia. However, the residual confounding from this would have to be greater than eightfold above and beyond the measured confounders in the high-dose ICS group and fourfold above and beyond the measured confounders in the medium-dose ICS group. We did not have information on medication adherence. For example, we assumed that patients prescribed SABAs more frequently used them more than those prescribed SABAs less frequently and that patients used all the doses within their ICS cannisters. However, misclassification from incorrectly assuming ICS adherence would bring the estimate toward the null, underrecognizing any adverse effects from ICSs. For this reason, calculating average daily dose was preferrable to using the first prescribed dose, as it accounted for all prescription doses and number of cannisters during follow-up. It is possible that some outcomes were misclassified, although this risk is low, as outcome events used hospital diagnoses only, for which there is easily available access to the necessary diagnostic tests. We did not have information on spirometry values.

### Conclusions

ICSs are instrumental in the management of asthma, reducing morbidity and mortality. Short-term use of low-dose ICSs was not associated with adverse outcomes (and has been shown to exert 80–90% of the benefits of ICSs [4, 5]). Medium- and high-dose ICSs were associated with an increased risk of adverse events, including MACEs, arrhythmia, PE, and pneumonia. The risk of each adverse event rose with increasing ICS dose. However, the overall absolute risk of each adverse outcome was low, about 20 times lower than the rate of asthma exacerbations in patients with mild asthma not using ICSs. Our findings therefore support current guideline recommendations to manage asthma at the minimally effective ICS dose and that corticosteroid stewardship and medication reviews should include high-dose ICS, and they emphasize the advantage of stepping down high-dose ICS, including in biologic-treated patients with severe asthma.

## Supplemental Materials

10.1164/rccm.202402-0368OC ONLINE DATA SUPPLEMENT
