# Supplementary material for: Association of Dose of Inhaled Corticosteroids and Frequency of Adverse Events
Source: Am J Respir Crit Care Med. 2024 Aug 1;211(1):54–63. doi: 10.1164/rccm.202402-0368OC (PMC11755354; doi:10.1164/rccm.202402-0368OC)
Supplement: ONLINE DATA SUPPLEMENT [file rccm.202402-0368OCS1.docx]

**Association of Dose of Inhaled Corticosteroids and Frequency of Adverse Events**

Chloë I Bloom, Freda Yang, Richard Hubbard, Azeem Majeed, Jadwiga A Wedzicha

**ONLINE DATA SUPPLEMENT**

STROBE Statement—checklist of items that should be included in reports of observational studies

|  | Item No. | Recommendation | Page  No. | Relevant text from manuscript |
| --- | --- | --- | --- | --- |
| **Title and abstract** | 1 | (*a*) Indicate the study’s design with a commonly used term in the title or the abstract | 1 |  |
|  |  | (*b*) Provide in the abstract an informative and balanced summary of what was done and what was found | 3 |  |
| Introduction | | | |  |
| Background/rationale | 2 | Explain the scientific background and rationale for the investigation being reported | 4 |  |
| Objectives | 3 | State specific objectives, including any prespecified hypotheses | 4-5 |  |
| Methods | | | |  |
| Study design | 4 | Present key elements of study design early in the paper | 5, 9-11 |  |
| Setting | 5 | Describe the setting, locations, and relevant dates, including periods of recruitment, exposure, follow-up, and data collection | 5 |  |
| Participants | 6 | (*a*) *Cohort study*—Give the eligibility criteria, and the sources and methods of selection of participants. Describe methods of follow-up  *Case-control study*—Give the eligibility criteria, and the sources and methods of case ascertainment and control selection. Give the rationale for the choice of cases and controls  *Cross-sectional study*—Give the eligibility criteria, and the sources and methods of selection of participants | 5-11 |  |
|  |  | (*b*) *Cohort study*—For matched studies, give matching criteria and number of exposed and unexposed  *Case-control study*—For matched studies, give matching criteria and the number of controls per case | 5-11 |  |
| Variables | 7 | Clearly define all outcomes, exposures, predictors, potential confounders, and effect modifiers. Give diagnostic criteria, if applicable | 5-7 |  |
| Data sources/ measurement | 8* | For each variable of interest, give sources of data and details of methods of assessment (measurement). Describe comparability of assessment methods if there is more than one group | 5 |  |
| Bias | 9 | Describe any efforts to address potential sources of bias | 7-11 |  |
| Study size | 10 | Explain how the study size was arrived at | Study protocol |  |

Continued on next page

| Quantitative variables | 11 | Explain how quantitative variables were handled in the analyses. If applicable, describe which groupings were chosen and why | 5-7 |  |
| --- | --- | --- | --- | --- |
| Statistical methods | 12 | (*a*) Describe all statistical methods, including those used to control for confounding | 7-11 |  |
|  |  | (*b*) Describe any methods used to examine subgroups and interactions | 7-11 |  |
|  |  | (*c*) Explain how missing data were addressed | 7-11 |  |
|  |  | (*d*) *Cohort study*—If applicable, explain how loss to follow-up was addressed  *Case-control study*—If applicable, explain how matching of cases and controls was addressed  *Cross-sectional study*—If applicable, describe analytical methods taking account of sampling strategy | 7-11 |  |
|  |  | (*e*) Describe any sensitivity analyses | 7-11 |  |
| Results | | | | |
| Participants | 13* | (a) Report numbers of individuals at each stage of study—eg numbers potentially eligible, examined for eligibility, confirmed eligible, included in the study, completing follow-up, and analysed | 11-13 |  |
|  |  | (b) Give reasons for non-participation at each stage | 11-13 |  |
|  |  | (c) Consider use of a flow diagram | 11-13 |  |
| Descriptive data | 14* | (a) Give characteristics of study participants (eg demographic, clinical, social) and information on exposures and potential confounders | 11-13 |  |
|  |  | (b) Indicate number of participants with missing data for each variable of interest | 11-13 |  |
|  |  | (c) *Cohort study*—Summarise follow-up time (eg, average and total amount) | 11-13 |  |
| Outcome data | 15* | *Cohort study*—Report numbers of outcome events or summary measures over time | 11-13 |  |
|  |  | *Case-control study—*Report numbers in each exposure category, or summary measures of exposure | 11-13 |  |
|  |  | *Cross-sectional study—*Report numbers of outcome events or summary measures |  |  |
| Main results | 16 | (*a*) Give unadjusted estimates and, if applicable, confounder-adjusted estimates and their precision (eg, 95% confidence interval). Make clear which confounders were adjusted for and why they were included | 11-13 |  |
|  |  | (*b*) Report category boundaries when continuous variables were categorized | 11-13 |  |
|  |  | (*c*) If relevant, consider translating estimates of relative risk into absolute risk for a meaningful time period | 11-13 |  |

Continued on next page

| Other analyses | 17 | Report other analyses done—eg analyses of subgroups and interactions, and sensitivity analyses | 11-13 |  |
| --- | --- | --- | --- | --- |
| Discussion | | | | |
| Key results | 18 | Summarise key results with reference to study objectives | 15 |  |
| Limitations | 19 | Discuss limitations of the study, taking into account sources of potential bias or imprecision. Discuss both direction and magnitude of any potential bias | 18-19 |  |
| Interpretation | 20 | Give a cautious overall interpretation of results considering objectives, limitations, multiplicity of analyses, results from similar studies, and other relevant evidence | 15-19 |  |
| Generalisability | 21 | Discuss the generalisability (external validity) of the study results | 15 |  |
| Other information | |  | | |
| Funding | 22 | Give the source of funding and the role of the funders for the present study and, if applicable, for the original study on which the present article is based | 1 |  |

The figures and tables are included in the page numbers shown, for example, flow diagram, study design, descriptive variables, unadjusted and adjusted results.

**Supplementary figures**

**Figure S1.** Study designs: **Main model:** ICS new-user cohort. **Secondary analyses models**: SCCS and case-control

**
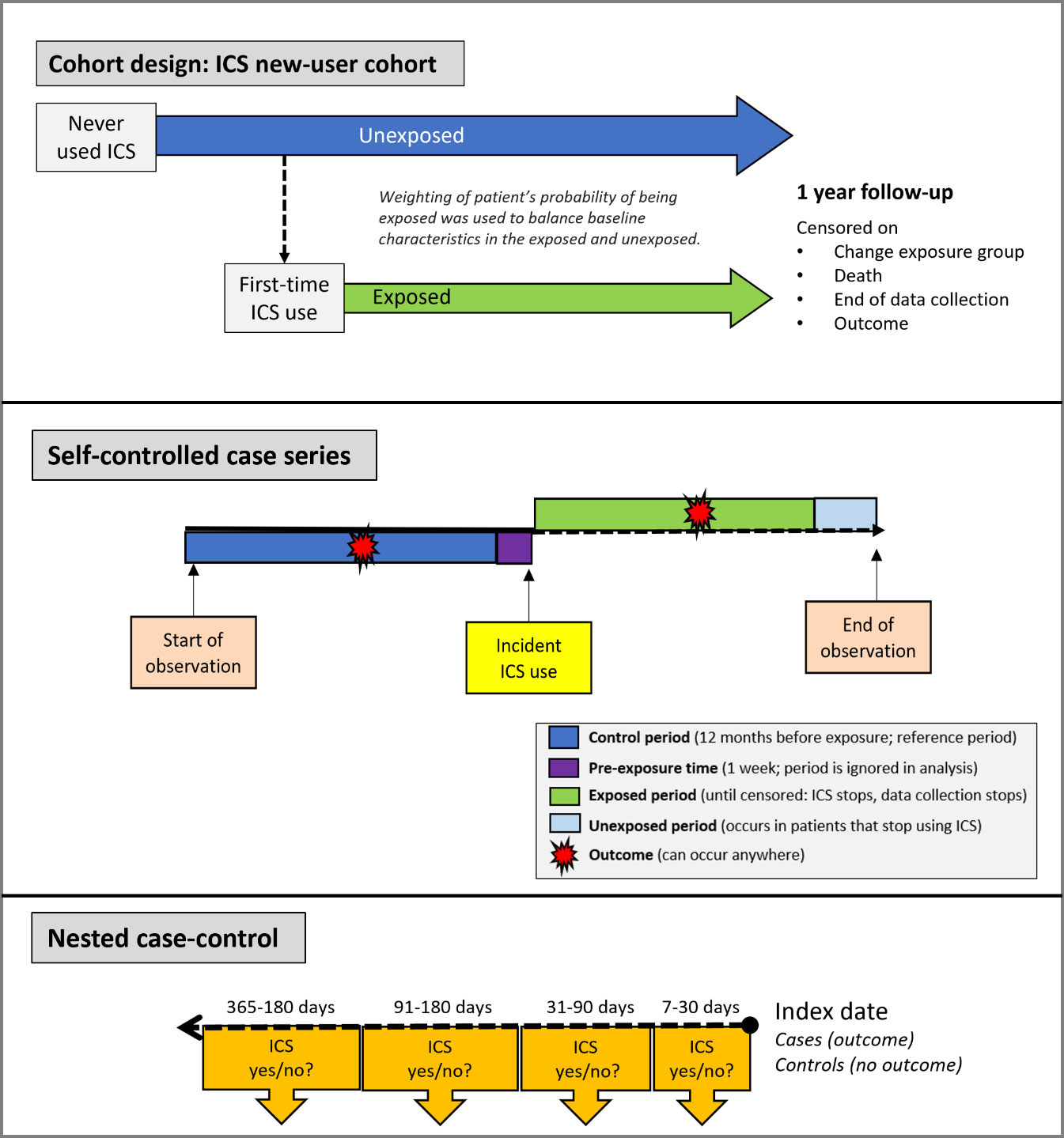
**

**Figure S2** Directed acyclic graphs for three main outcomes (CVD, pneumonia and PE)


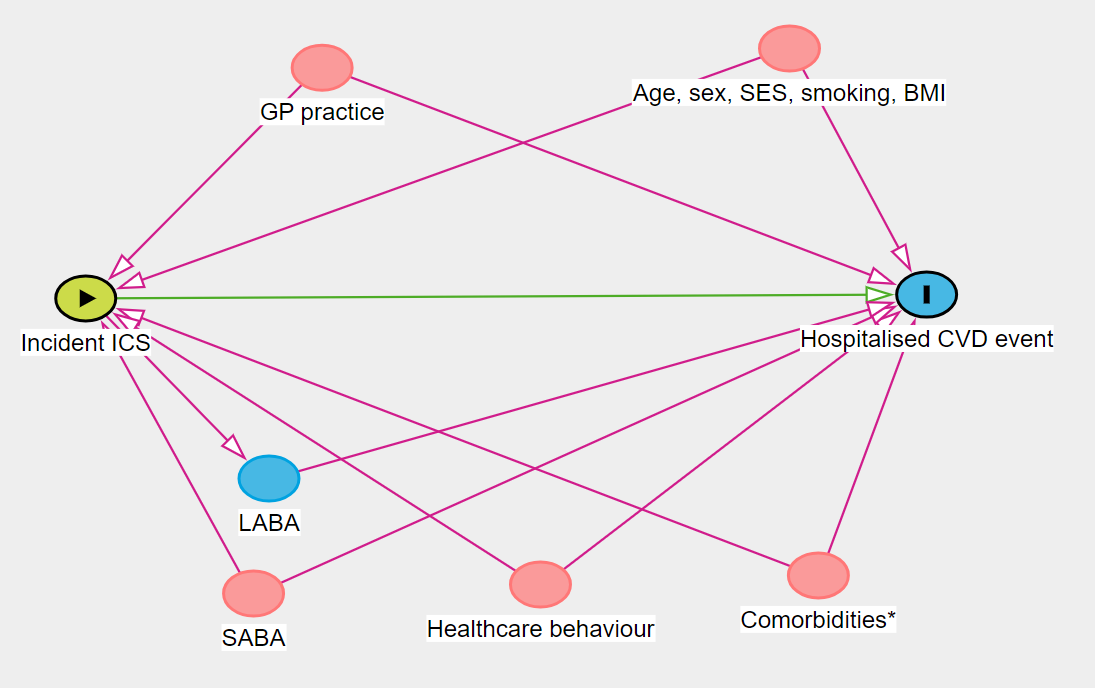


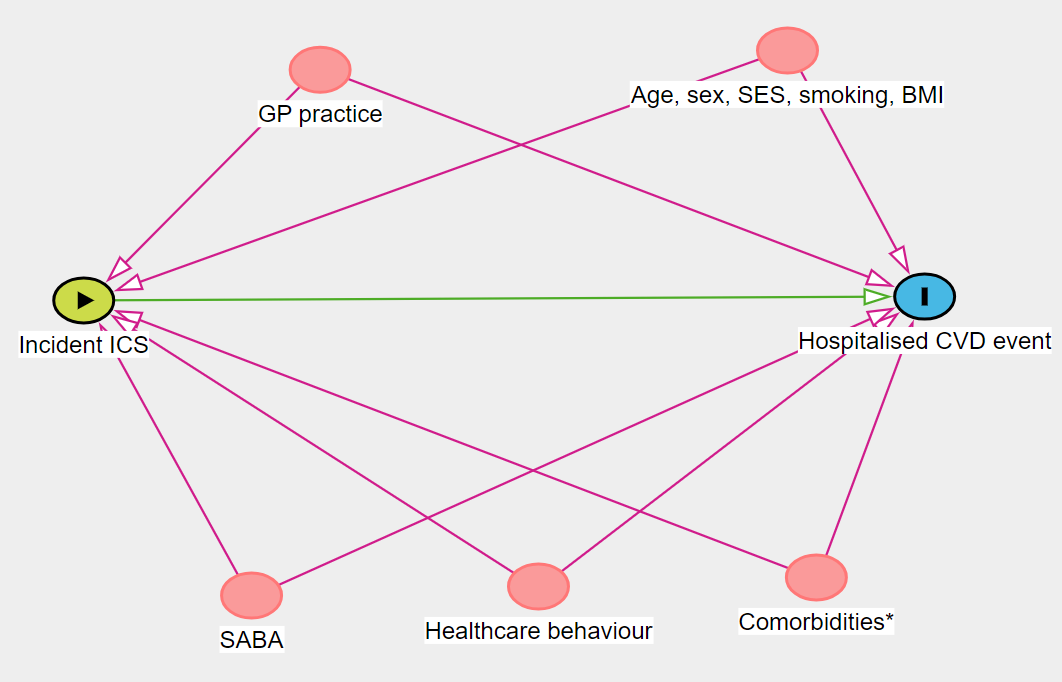


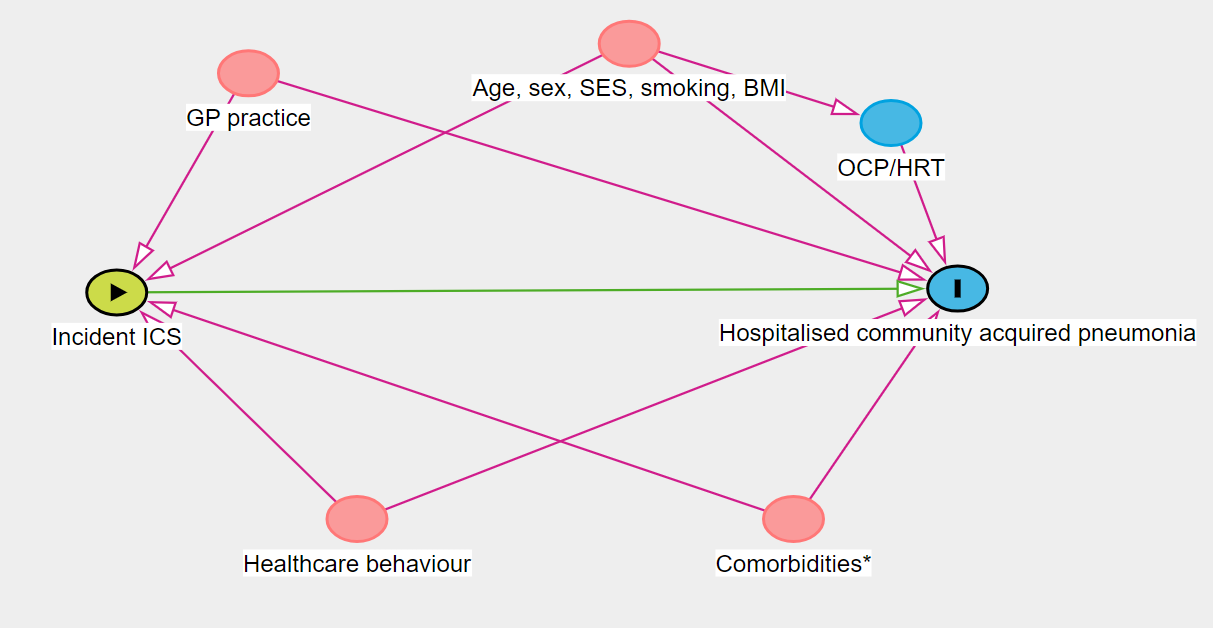


*comorbidities include cardiovascular disease (CVD), chronic obstructive pulmonary disease, type-2 diabetes, chronic renal failure, anxiety, depression (and cancer for PE outcome). SES=socioeconomic status, BMI=body mass index, OCP=oral contraceptive pill, HRT=hormone replacement therapy, ICS=inhaled corticosteroid, GP=general practitioner

**Figure S3.** Flow diagram of study inclusion and exclusion


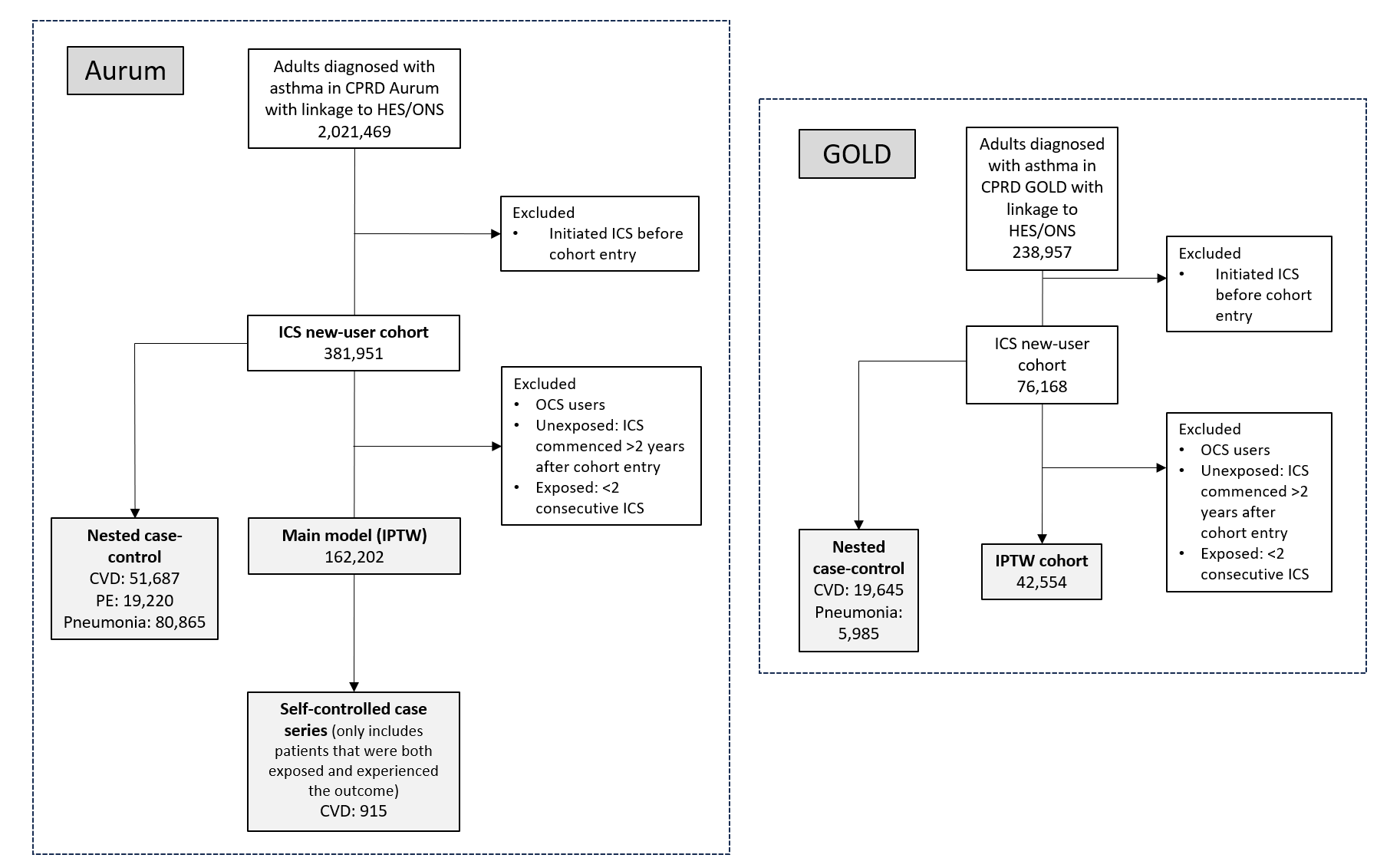


**Figure S4.** Kaplan Meier plots for each outcome


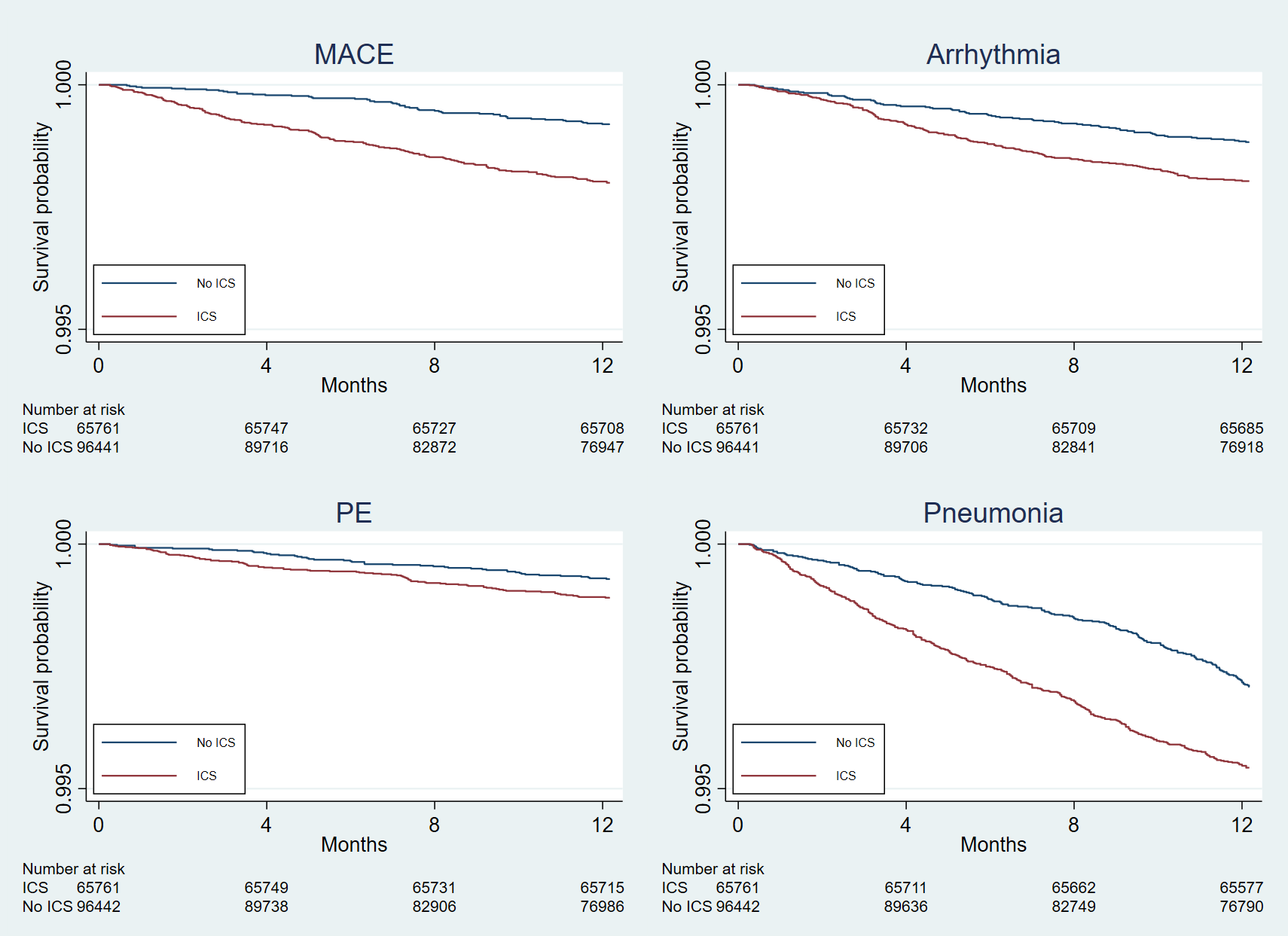


**Figure S5.** Association between ICS and outcomes, by first ICS dose prescribed (main cohort)


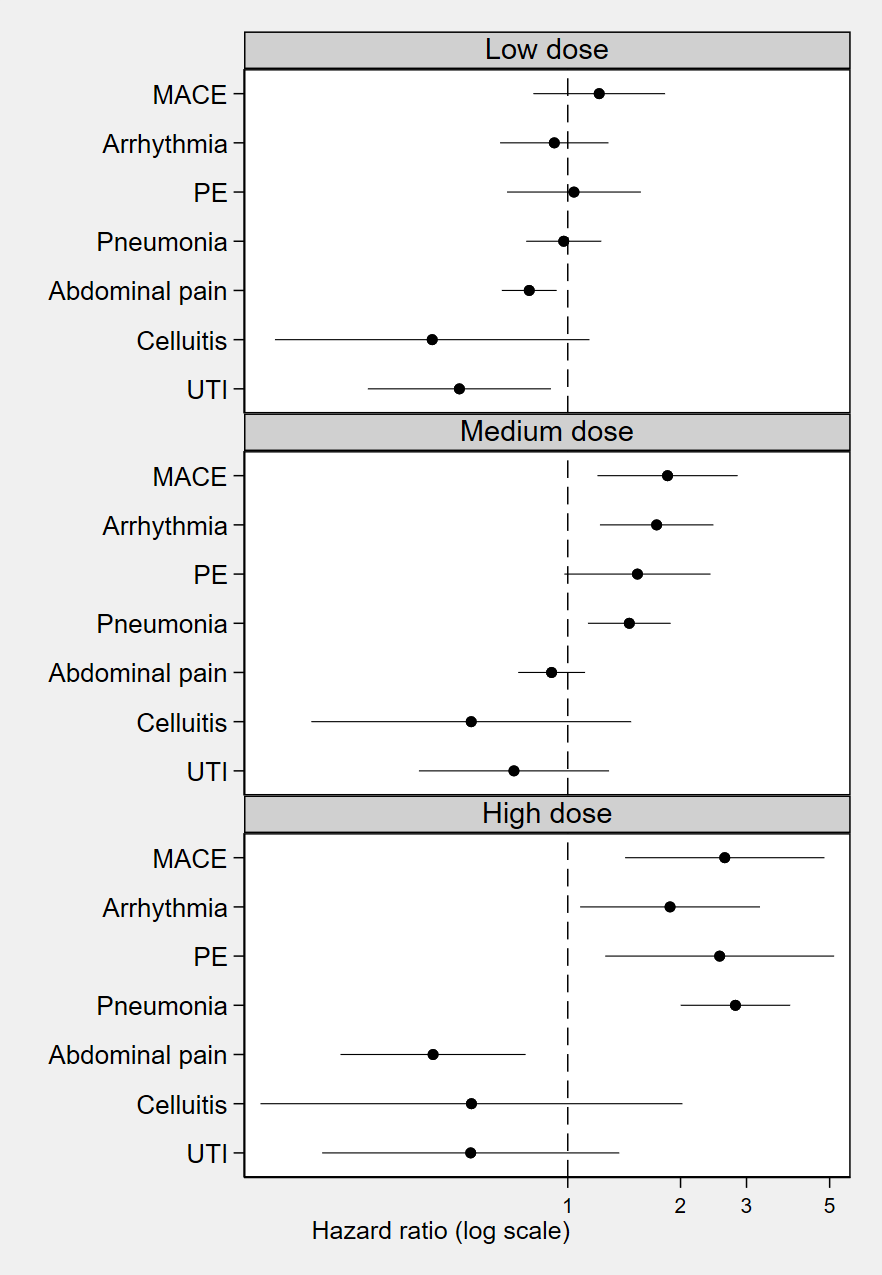


MACE=major adverse cardiovascular event, UTI=urinary tract infection, PE=pulmonary embolism

**Figure S6.** Association between ICS and outcomes, CVD and pneumonia, by categorised average daily ICS dose (secondary analysis: GOLD IPTW cohort)

**
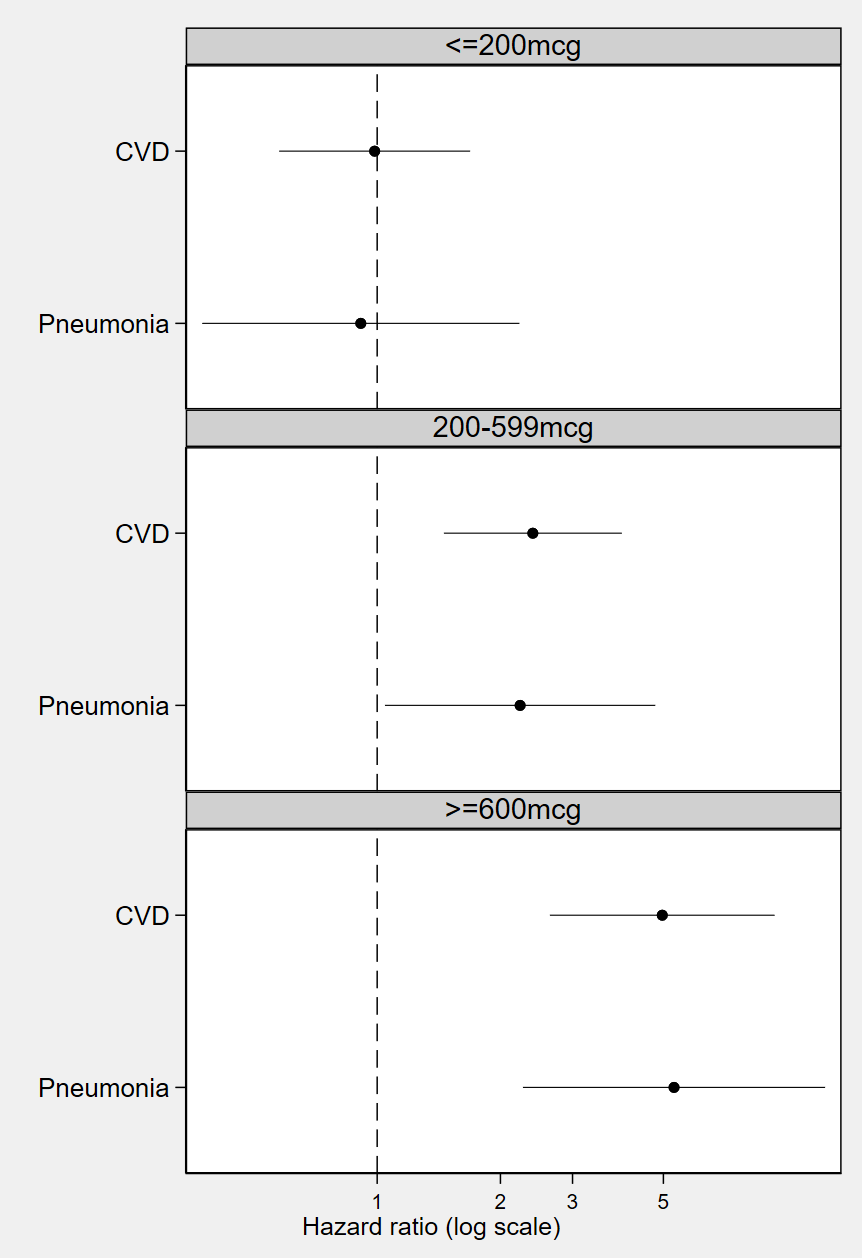
**

**Figure S7.** Negative control analysis showing the association between citalopram and outcomes by time since last ICS prescription (main analysis: Aurum IPTW cohort)


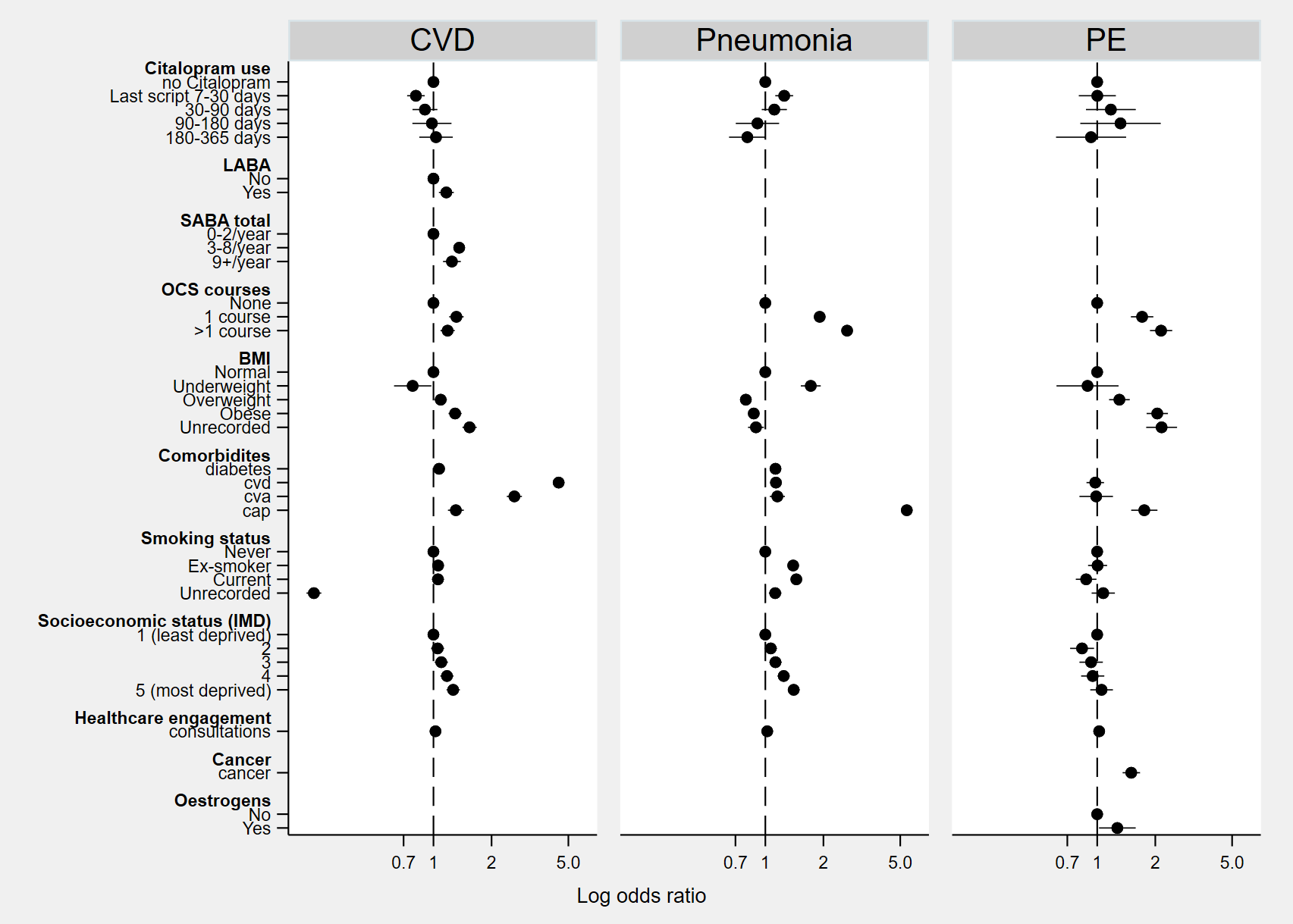


CVD=cardiovascular disease, PE=pulmonary embolism, LABA=long-acting beta-agonist, SABA=short-acting beta-agonist, OCS=oral corticosteroids, BMI=body mass index, IMD=index multiple deprivation, ICS=inhaled corticosteroids.

**Figure S8**. Association between CVD and ICS and citalopram, by time since last prescription (secondary analysis: GOLD nested case-control - CVD)


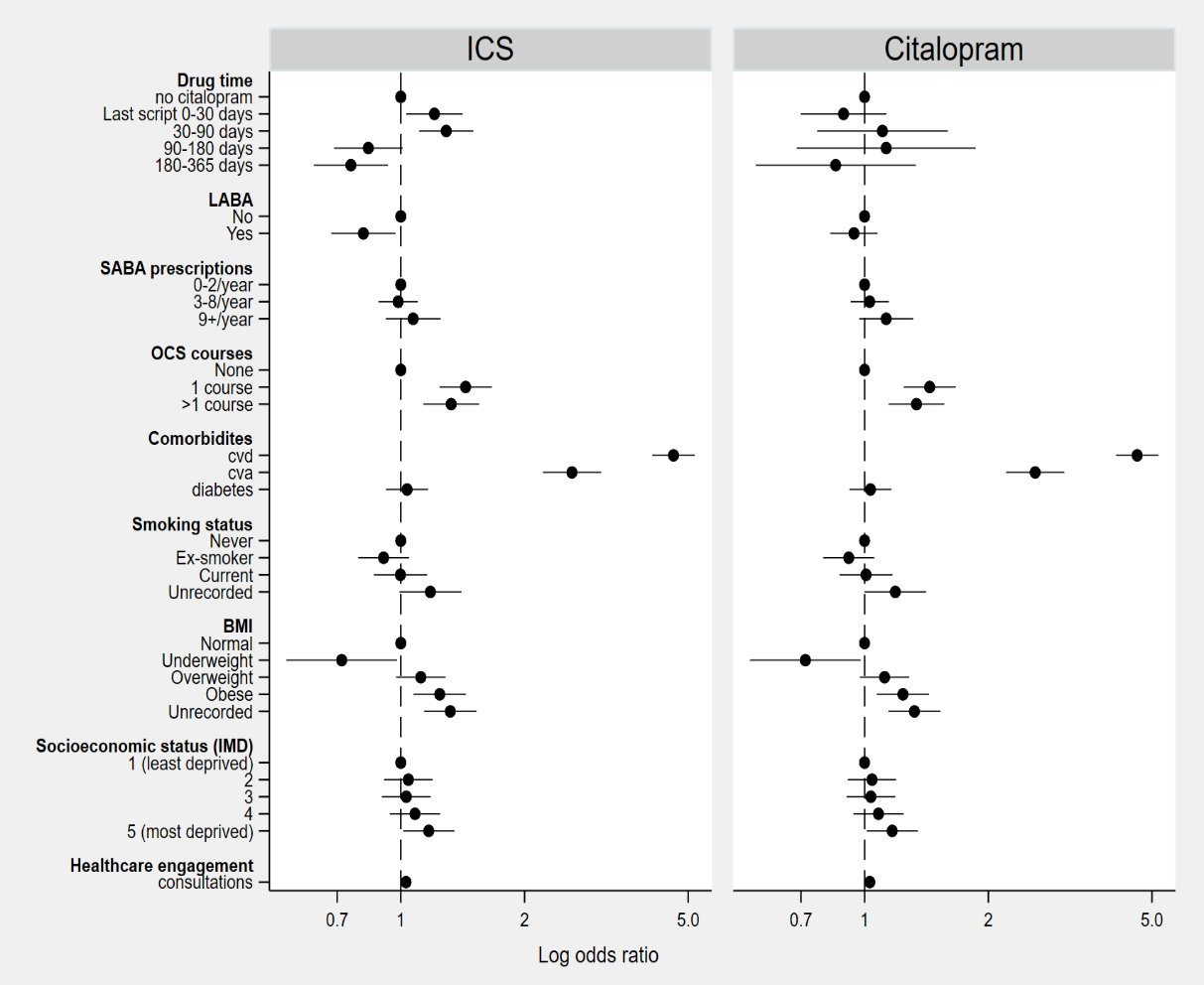


ICS=inhaled corticosteroids, LABA=long-acting beta-agonist, SABA=short-acting beta-agonist, OCS=oral corticosteroids, BMI=body mass index, IMD=index multiple deprivation

**Figure S9**. Association between pneumonia and ICS, by time since last ICS prescription (secondary analysis: GOLD nested case-control - pneumonia).


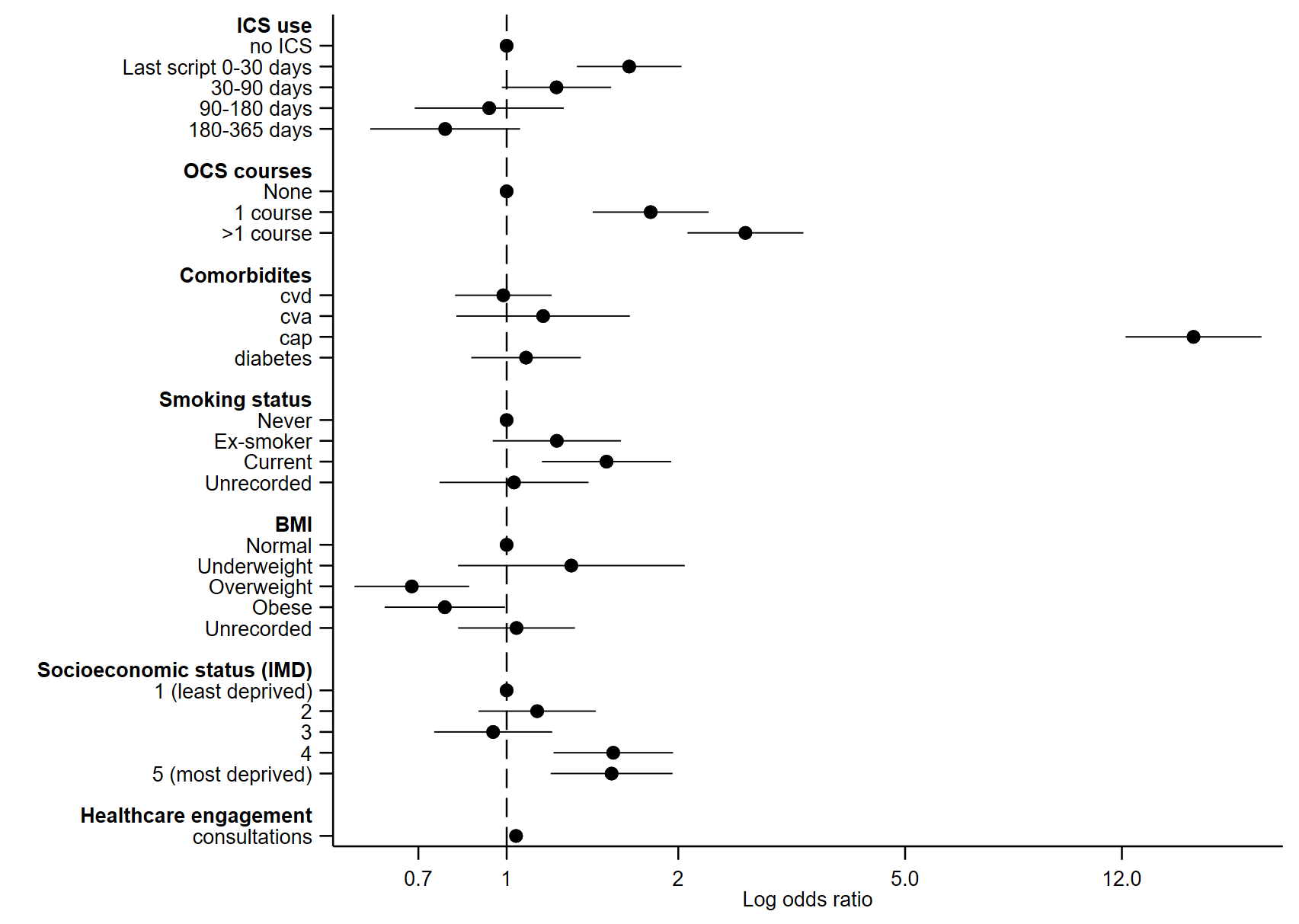


**Figure S10**. Association between ICS and each outcome, stratified by the variables shown.


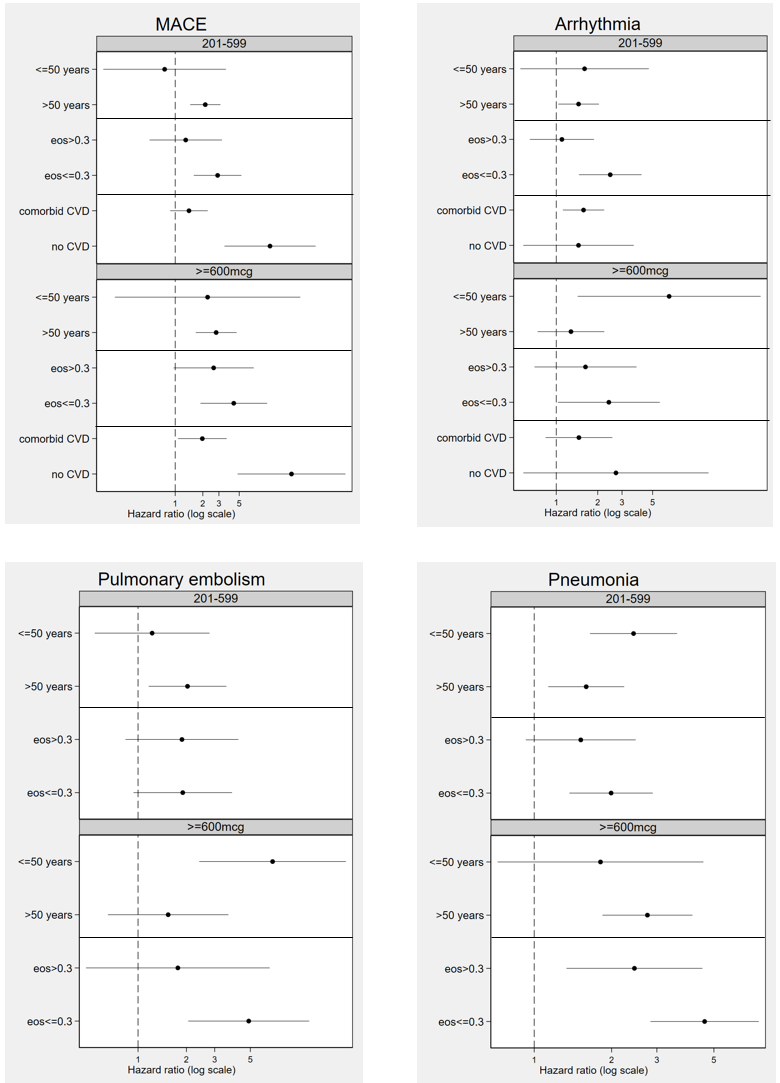


**Figure S11.** Association with each outcome, budesonide compared to beclomethasone, and fluticasone compared to beclomethasone, in the IPTW new-user cohort additionally accounting for ICS dose.


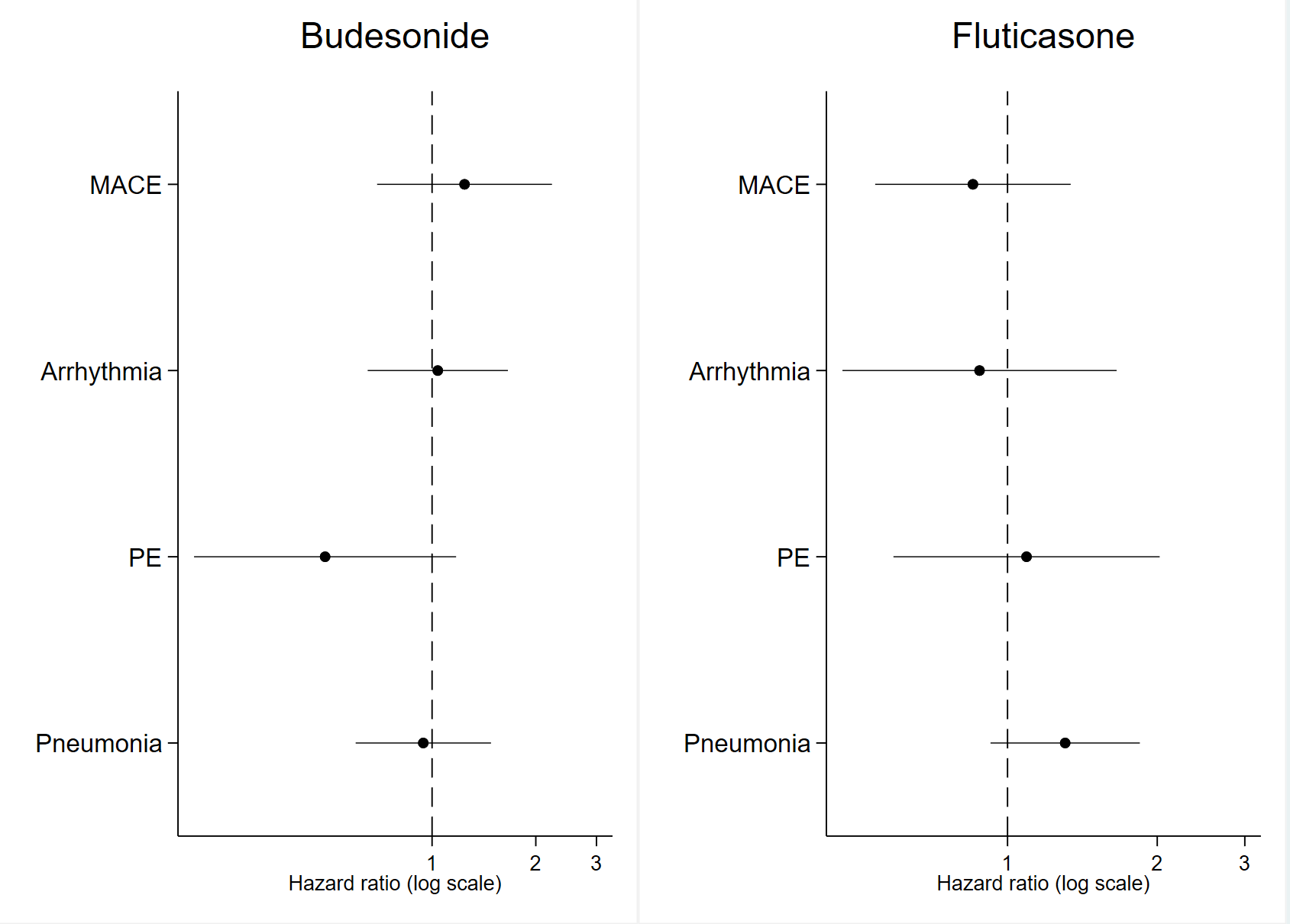


1. **Supplementary tables**
2. Contents

[Table S1. Characteristics of Aurum ICS new-user cohort before and after weighting 41](#_Toc162275227)

[Table S2. Rates of outcomes (Aurum ICS new-user cohort) 42](#_Toc162275228)

[Table S3. Association between ICS and each adverse outcome, by categorised average daily ICS use (Aurum ICS new-user cohort). 44](#_Toc162275229)

[Table S4. E-values for each outcome in the main analysis that was found to be significantly associated with ICS 45](#_Toc162275230)

[Table S5. Characteristics of GOLD ICS new-user cohort before and after weighting 46](#_Toc162275231)

[Table S6. Association between ICS and CVD (Aurum nested case-control) 48](#_Toc162275232)

[Table S7. Association between ICS and hospitalised pneumonia (Aurum nested case-control) 49](#_Toc162275233)

[Table S8. Association between ICS and PE (Aurum nested case-control) 51](#_Toc162275234)

[Table S9. Association between ICS and CVD (GOLD nested case-control) 52](#_Toc162275235)

[Table S10. Association between ICS and hospitalised pneumonia (GOLD nested case-control) 53](#_Toc162275236)

[Table S11. Association between ICS and CVD (Aurum SCCS) 54](#_Toc162275237)

[Table S12. Association between fluticasone compared to beclomethasone, and budesonide compared to beclomethasone (Aurum ICS new-user cohort) 55](#_Toc162275238)

- Table S1. Characteristics of Aurum ICS new-user cohort before and after weighting

| **Variables** | **Before weighting** | | | | | **After weighting** | | | | |
| --- | --- | --- | --- | --- | --- | --- | --- | --- | --- | --- |
|  | **ICS** | | **no ICS** | | ***SD*** | **ICS** | | **no ICS** | | ***SD*** |
|  | N | **%** | N | **%** |  | N | **%** | N | **%** |  |
| **Total** | 96,441 | 59.5% | 65,761 | 40.5% |  | 96,441 | 59.5% | 65,761 | 40.5% |  |
| Mean age (SD), years | 43.0 (19.8) | | 37.7 (17.9) | | *0.279* | 40.5 (19.9) | | 40.5 (9.7) | | *0.005* |
| Males | 41,277 | 42.8% | 28,475 | 43.3% | *0.009* | 41,663 | 43.2% | 28,409 | 43.2% | *<0.0001* |
| **IMD** |  |  |  |  |  |  |  |  |  |  |
| 1 (least deprived) | 17,649 | 18.3% | 12,692 | 19.3% | *0.025* | 17,938 | 18.6% | 12,232 | 18.6% | *0.001* |
| 2 | 18,806 | 19.5% | 13,152 | 20.0% | *0.013* | 18,999 | 19.7% | 12,823 | 19.5% | *0.004* |
| 3 | 18,420 | 19.1% | 12,692 | 19.3% | *0.005* | 18,517 | 19.2% | 12,692 | 19.3% | *0.004* |
| 4 | 20,253 | 21.0% | 13,876 | 21.1% | *0.002* | 20,349 | 21.1% | 13,876 | 21.1% | *0.001* |
| 5 | 21,313 | 22.1% | 13,349 | 20.3% | *0.044* | 20,638 | 21.4% | 14,073 | 21.4% | *0.001* |
| **BMI** |  |  |  |  |  |  |  |  |  |  |
| Normal | 27,679 | 28.7% | 21,701 | 33.0% | *0.095* | 29,704 | 30.8% | 20,320 | 30.9% | *0.002* |
| Underweight | 3,568 | 3.7% | 2,565 | 3.9% | *0.011* | 3,665 | 3.8% | 2,499 | 3.8% | *0.002* |
| Overweight | 36,744 | 38.1% | 24,332 | 37.0% | *0.022* | 36,165 | 37.5% | 24,595 | 37.4% | *0.002* |
| Obese | 23,242 | 24.1% | 13,021 | 19.8% | *0.105* | 21,313 | 22.1% | 14,467 | 22.0% | *0.001* |
| Unrecorded | 5,304 | 5.5% | 4,143 | 6.3% | *0.036* | 5,690 | 5.9% | 3,880 | 5.9% | *0.002* |
| **Smoking** |  |  |  |  |  |  |  |  |  |  |
| Never | 41,373 | 42.9% | 28,935 | 44.0% | *0.023* | 42,241 | 43.8% | 28,869 | 43.9% | *0.002* |
| Ex-smoker | 23,532 | 24.4% | 17,295 | 26.3% | *0.043* | 24,400 | 25.3% | 16,769 | 25.5% | *0.003* |
| Current | 20,445 | 21.2% | 9,996 | 15.2% | *0.154* | 17,938 | 18.6% | 12,100 | 18.4% | *0.005* |
| Unrecorded | 11,091 | 11.5% | 9,535 | 14.5% | *0.087* | 11,862 | 12.3% | 8,089 | 12.3% | *0.001* |
| **Comorbidities** |  |  |  |  |  |  |  |  |  |  |
| CVD | 23,435 | 24.3% | 13,678 | 20.8% | *0.083* | 21,892 | 22.7% | 14,928 | 22.7% | *0.001* |
| Hospitalised CVD | 1,350 | 1.4% | 526 | 0.8% | *0.057* | 1,061 | 1.1% | 789 | 1.2% | *0.006* |
| CVA | 1,736 | 1.8% | 921 | 1.4% | *0.029* | 1,543 | 1.6% | 1,052 | 1.6% | *<0.0001* |
| COPD | 10,319 | 10.7% | 6,247 | 9.5% | *0.040* | 9,837 | 10.2% | 6,839 | 10.4% | *0.006* |
| Type-2 diabetes | 11,766 | 12.2% | 7,102 | 10.8% | *0.044* | 11,187 | 11.6% | 7,694 | 11.7% | *0.004* |
| Chronic renal failure | 5,304 | 5.5% | 3,157 | 4.8% | *0.033* | 5,015 | 5.2% | 3,420 | 5.2% | *0.001* |
| Depression | 12,923 | 13.4% | 10,522 | 16.0% | *0.074* | 13,984 | 14.5% | 9,535 | 14.5% | *<0.0001* |
| Previous pneumonia | 1,832 | 1.9% | 1,381 | 2.1% | *0.015* | 1,929 | 2.0% | 1,315 | 2.0% | *<0.0001* |
| Anxiety | 10,898 | 11.3% | 8,746 | 13.3% | *0.062* | 11,669 | 12.1% | 7,957 | 12.1% | *0.001* |
| **SABA cannisters (year before)** | |  |  |  |  |  |  |  |  |  |
| 0-2 | 84,868 | 88.0% | 60,106 | 91.4% | *0.111* | 85,929 | 89.1% | 58,790 | 89.4% | *0.007* |
| 3-7 | 8,583 | 8.9% | 4,143 | 6.3% | *0.096* | 7,908 | 8.2% | 4,866 | 7.4% | *0.032* |
| ≥8 | 2,990 | 3.1% | 1,513 | 2.3% | *0.051* | 2,604 | 2.7% | 1,776 | 2.7% | *0.037* |

CVD=cardiovascular disease, CVA=cerebrovascular accident, SABA=short-acting beta-agonist, BMI=body mass index, IMD=index multiple deprivation, SD=standardised difference, ICS=inhaled corticosteroids

- Table S2. Rates of outcomes (Aurum ICS new-user cohort)

| **Patients** | **Events** | **Years (1000)** | **Rates*** | **LCI 95%** | **UCI 95%** |
| --- | --- | --- | --- | --- | --- |
| **MACE** |  |  |  |  |  |
| **ALL** | 229 | 152.2 | 1.5 | 1.3 | 1.7 |
| **Average daily dose categorised** | | |  |  |  |
| **None** | 53 | 65.7 | 0.8 | 0.6 | 1.1 |
| **<=200mcg** | 55 | 57.2 | 1.0 | 0.7 | 1.3 |
| **201-599** | 96 | 25.3 | 3.8 | 3.1 | 4.6 |
| **>=600mcg** | 25 | 4.0 | 6.3 | 4.2 | 9.3 |
| **First ICS inhaler dose** | |  |  |  |  |
| **None** | 53 | 65.7 | 0.8 | 0.6 | 1.1 |
| **Low dose** | 97 | 56.3 | 1.7 | 1.4 | 2.1 |
| **Medium dose** | 63 | 25.8 | 2.4 | 1.9 | 3.1 |
| **High dose** | 16 | 4.4 | 3.6 | 2.2 | 5.9 |
| **Hospitalised arrhythmia** | |  |  |  |  |
| **ALL** | 250 | 152.2 | 1.6 | 1.5 | 1.9 |
| **Average daily dose categorised** | | |  |  |  |
| **None** | 77 | 65.7 | 1.2 | 0.9 | 1.5 |
| **<=200mcg** | 65 | 57.2 | 1.1 | 0.9 | 1.4 |
| **201-599** | 90 | 25.3 | 3.6 | 2.9 | 4.4 |
| **>=600mcg** | 18 | 4.0 | 4.5 | 2.8 | 7.2 |
| **First ICS inhaler dose** | |  |  |  |  |
| **None** | 77 | 65.7 | 1.2 | 0.9 | 1.5 |
| **Low dose** | 87 | 56.3 | 1.5 | 1.3 | 1.9 |
| **Medium dose** | 70 | 25.8 | 2.7 | 2.1 | 3.4 |
| **High dose** | 16 | 4.4 | 3.6 | 2.2 | 6.0 |
| **Hospitalised pneumonia** | |  |  |  |  |
| **ALL** | 592 | 152.1 | 3.9 | 3.6 | 4.2 |
| **Average daily dose categorised** | | |  |  |  |
| **None** | 192 | 65.7 | 2.9 | 2.5 | 3.4 |
| **<=200mcg** | 130 | 57.2 | 2.3 | 1.9 | 2.7 |
| **201-599** | 207 | 25.2 | 8.2 | 7.2 | 9.4 |
| **>=600mcg** | 63 | 4.0 | 15.8 | 12.4 | 20.3 |
| **First ICS inhaler dose** | |  |  |  |  |
| **None** | 192 | 65.7 | 2.9 | 2.5 | 3.4 |
| **Low dose** | 130 | 57.2 | 2.3 | 1.9 | 2.7 |
| **Medium dose** | 207 | 25.2 | 8.2 | 7.2 | 9.4 |
| **High dose** | 63 | 4.0 | 15.8 | 12.4 | 20.3 |
| **Pulmonary embolism** | |  |  |  |  |
| **ALL** | 143 | 152.3 | 0.9 | 0.8 | 1.1 |
| **Average daily dose categorised** | | |  |  |  |
| **None** | 47 | 65.7 | 0.7 | 0.5 | 1.0 |
| **<=200mcg** | 41 | 57.2 | 0.7 | 0.5 | 1.0 |
| **201-599** | 44 | 25.3 | 1.7 | 1.3 | 2.3 |
| **>=600mcg** | 11 | 4.0 | 2.8 | 1.5 | 5.0 |
| **First ICS inhaler dose** | |  |  |  |  |
| **None** | 47 | 65.7 | 0.7 | 0.5 | 1.0 |
| **Low dose** | 41 | 57.2 | 0.7 | 0.5 | 1.0 |
| **Medium dose** | 44 | 25.3 | 1.7 | 1.3 | 2.3 |
| **High dose** | 11 | 4.0 | 2.8 | 1.5 | 5.0 |

- Table S3. Association between ICS and each adverse outcome, by categorised average daily ICS use (Aurum ICS new-user cohort).

| **ICS average daily dose** | **Adverse outcome** | **Unadjusted, unweighted HR** | **95% CI** | **p-value** | **Weighted HR** | **95% CI** | **p-value** |
| --- | --- | --- | --- | --- | --- | --- | --- |
| **≤200mcg** | **MACE** | 1.19 | 0.81-1.73 | 0.373 | 0.66 | 0.40-1.62 | 0.090 |
|  | **Arrhythmia** | 0.96 | 0.69-1.33 | 0.815 | 0.64 | 0.45-0.90 | 0.011 |
|  | **PE** | 0.99 | 0.66-1.51 | 0.997 | 0.85 | 0.54-1.32 | 0.470 |
|  | **Pneumonia** | 0.78 | 0.62-0.97 | 0.028 | 0.62 | 0.47-0.80 | <0.0001 |
| **201-599mcg** | **MACE** | 4.66 | 3.32-6.51 | <0.0001 | 2.63 | 1.66-4.15 | <0.0001 |
|  | **Arrhythmia** | 2.99 | 2.20-4.05 | <0.0001 | 2.21 | 1.60-3.04 | <0.0001 |
|  | **PE** | 2.41 | 1.59-3.63 | <0.0001 | 2.10 | 1.37-3.22 | 0.001 |
|  | **Pneumonia** | 2.81 | 2.31-3.42 | <0.0001 | 2.25 | 1.77-2.85 | <0.0001 |
| **≥600mcg** | **MACE** | 7.69 | 4.77-12.38 | <0.0001 | 4.63 | 2.62-8.17 | <0.0001 |
|  | **Arrhythmia** | 3.81 | 2.28-6.37 | <0.0001 | 2.91 | 1.72-4.91 | <0.0001 |
|  | **PE** | 3.81 | 1.98-7.35 | <0.0001 | 3.32 | 1.69-6.50 | <0.0001 |
|  | **Pneumonia** | 5.43 | 4.08-7.23 | <0.0001 | 4.09 | 2.98-5.60 | <0.0001 |

- Table S4. E-values for each outcome in the main analysis that was found to be significantly associated with ICS

|  |  | **e-value** | |
| --- | --- | --- | --- |
| **Outcome** | **ICS dose** | **Point estimate** | **Confidence interval** |
| **MACE** | **Medium** | 4.68 | 2.71 |
|  | **High** | 8.73 | 4.68 |
| **Arrhythmia** | **Medium** | 3.85 | 2.58 |
|  | **High** | 5.27 | 2.83 |
| **PE** | **Medium** | 3.62 | 2.08 |
|  | **High** | 6.10 | 2.77 |
| **Pneumonia** | **Medium** | 3.93 | 2.94 |
|  | **High** | 7.65 | 5.41 |

- Table S5. Characteristics of GOLD ICS new-user cohort before and after weighting

|  | **Unweighted** | | | | | **Weighted** | | | | |
| --- | --- | --- | --- | --- | --- | --- | --- | --- | --- | --- |
|  | **ICS** | | **no ICS** | | **SD** | **ICS** | | **no ICS** | | **SD** |
|  | **N** | **%** | **N** | **%** |  | **N** | **%** | **N** | **%** |  |
| **Total** | 21,955 | 51.6 | 20,599 | 48.4 |  | 21,955 | 51.6 | 20,599 | 48.4 |  |
| Mean age (SD), years | 42.3 (17.7) | | 36.8 (16.8) | | 0.317 | 39.45 (16.9) | | 40.28 (18.5) | | 0.049 |
| Males | 9,111 | 41.5% | 8,858 | 43.0% | 0.030 | 9,243 | 42.1% | 8,816 | 42.8% | 0.016 |
| **IMD** |  |  |  |  |  |  |  |  |  |  |
| 1 (least deprived) | 5,116 | 23.3% | 4,923 | 23.9% | 0.014 | 5,181 | 23.6% | 4,923 | 23.9% | 0.006 |
| 2 | 4,742 | 21.6% | 4,367 | 21.2% | 0.010 | 4,676 | 21.3% | 4,429 | 21.5% | 0.003 |
| 3 | 4,435 | 20.2% | 4,140 | 20.1% | 0.003 | 4,457 | 20.3% | 4,223 | 20.5% | 0.003 |
| 4 | 4,040 | 18.4% | 3,831 | 18.6% | 0.006 | 4,040 | 18.4% | 3,749 | 18.2% | 0.006 |
| 5 | 3,623 | 16.5% | 3,337 | 16.2% | 0.009 | 3,579 | 16.3% | 3,296 | 16.0% | 0.009 |
| **BMI** |  |  |  |  |  |  |  |  |  |  |
| Normal | 4,391 | 20.0% | 4,552 | 22.1% | 0.052 | 4,654 | 21.2% | 4,346 | 21.1% | 0.003 |
| Underweight | 725 | 3.3% | 989 | 4.8% | 0.079 | 900 | 4.1% | 824 | 4.0% | 0.003 |
| Overweight | 6,213 | 28.3% | 5,109 | 24.8% | 0.079 | 5,862 | 26.7% | 5,582 | 27.1% | 0.010 |
| Obese | 4,084 | 18.6% | 3,275 | 15.9% | 0.072 | 3,754 | 17.1% | 3,502 | 17.0% | <0.001 |
| Unrecorded | 6,543 | 29.8% | 6,653 | 32.3% | 0.055 | 6,806 | 31.0% | 6,324 | 30.7% | 0.006 |
| **Smoking** |  |  |  |  |  |  |  |  |  |  |
| Never | 7,487 | 34.1% | 7,539 | 36.6% | 0.051 | 7,816 | 35.6% | 7,230 | 35.1% | 0.010 |
| Ex-smoker | 4,633 | 21.1% | 2,595 | 12.6% | 0.229 | 3,710 | 16.9% | 3,667 | 17.8% | 0.025 |
| Current | 3,140 | 14.3% | 3,605 | 17.5% | 0.087 | 3,469 | 15.8% | 3,152 | 15.3% | 0.012 |
| Unrecorded | 6,696 | 30.5% | 6,880 | 33.4% | 0.063 | 6,960 | 31.7% | 6,530 | 31.7% | 0.001 |
| **Comorbidities** |  |  |  |  |  |  |  |  |  |  |
| CVD | 3,579 | 16.3% | 2,348 | 11.4% | 0.144 | 3,030 | 13.8% | 2,925 | 14.2% | 0.011 |
| Hospitalised CVD | 307 | 1.4% | 165 | 0.8% | 0.056 | 242 | 1.1% | 247 | 1.2% | 0.003 |
| CVA | 176 | 0.8% | 103 | 0.5% | 0.046 | 132 | 0.6% | 165 | 0.8% | 0.022 |
| COPD | 593 | 2.7% | 247 | 1.2% | 0.108 | 439 | 2.0% | 515 | 2.5% | 0.035 |
| Type-2 diabetes | 944 | 4.3% | 577 | 2.8% | 0.082 | 790 | 3.6% | 783 | 3.8% | 0.012 |
| Chronic renal failure | 527 | 2.4% | 144 | 0.7% | 0.139 | 373 | 1.7% | 391 | 1.9% | 0.013 |
| Depression | 3,315 | 15.1% | 2,719 | 13.2% | 0.056 | 3,162 | 14.4% | 2,966 | 14.4% | 0.002 |
| Previous pneumonia | 483 | 2.2% | 536 | 2.6% | 0.031 | 527 | 2.4% | 474 | 2.3% | 0.004 |
| Anxiety | 88 | 0.4% | 62 | 0.3% | 0.017 | 88 | 0.4% | 82 | 0.4% | 0.001 |
| **SABA cannisters (year before)** | |  |  |  |  |  |  |  |  |  |
| 0-2 | 17,169 | 78.2% | 20,043 | 97.3% | 0.609 | 19,298 | 87.9% | 17,674 | 85.8% | 0.061 |
| ≥2 | 4,786 | 21.8% | 556 | 2.7% | 0.609 | 2,657 | 12.1% | 2,925 | 14.2% | 0.061 |

- Table S6. Association between ICS and CVD (Aurum nested case-control)

| **Variable** | **Non-ICS users** | | **ICS users** | | **Adjusted OR** | **p-value** | **95% CI** |
| --- | --- | --- | --- | --- | --- | --- | --- |
|  | **N** | **%** | **N** | **%** |  |  |  |
| **Total** | 30,532 | 59.1 | 21,155 | 40.9 |  |  |  |
| **Mean age (SD)** | 62.3 (15.5) | | 63.6 (14.2) | |  |  |  |
| **Females** | 14,629 | 47.9 | 10,316 | 48.8 |  |  |  |
| **Last ICS** |  |  |  |  |  |  |  |
| No ICS | 30,532 | 100.0 | 0 | 0.0 | Reference |  |  |
| 7-30 days | 0 | 0.0 | 6,818 | 32.2 | 1.60 | <0.0001 | 1.44-1.77 |
| 31-90 days | 0 | 0.0 | 6,550 | 31.0 | 1.52 | <0.0001 | 1.40-1.64 |
| 91-180 days | 0 | 0.0 | 3,412 | 16.1 | 1.21 | <0.0001 | 1.09-1.34 |
| 181-365 days | 0 | 0.0 | 4,375 | 20.7 | 1.02 | 0.618 | 0.93-1.13 |
| **LABA use** | 0 | 0.0 | 4,136 | 19.6 | 0.90 | 0.095 | 0.80-1.02 |
| **SABA in last year** |  |  |  |  |  |  |  |
| 0-3 | 28,324 | 92.8 | 11,918 | 56.3 | Reference |  |  |
| 4-8 | 1,738 | 5.7 | 6,866 | 32.5 | 1.17 | <0.0001 | 1.09-1.25 |
| >8 | 470 | 1.5 | 2,371 | 11.2 | 1.05 | 0.354 | 0.94-1.17 |
| **OCS in last year** |  |  |  |  |  |  |  |
| None | 27,757 | 90.9 | 15,603 | 73.8 | Reference |  |  |
| 1 course | 1,342 | 4.4 | 2,665 | 12.6 | 1.26 | <0.0001 | 1.16-1.38 |
| >1 course | 1,433 | 4.7 | 2,887 | 13.7 | 1.15 | 0.002 | 1.05-1.25 |
| **BMI** |  |  |  |  |  |  |  |
| Normal | 5,718 | 18.7 | 4,234 | 20.0 | Reference |  |  |
| Underweight | 447 | 1.5 | 424 | 2.0 | 0.79 | 0.035 | 0.63-0.98 |
| Overweight | 11,036 | 36.2 | 8,157 | 38.6 | 1.09 | 0.021 | 1.01-1.17 |
| Obese | 6,573 | 21.5 | 5,517 | 26.1 | 1.29 | <0.0001 | 1.19-1.40 |
| Unrecorded | 6,758 | 22.1 | 2,823 | 13.3 | 1.56 | <0.0001 | 1.43-1.69 |
| **Diabetes** | 5,859 | 19.2 | 4,796 | 22.7 | 1.08 | 0.012 | 1.02-1.15 |
| **CVD** | 13,647 | 44.7 | 13,754 | 65.0 | 4.46 | <0.0001 | 4.17-4.77 |
| **CVA** | 1,515 | 5.0 | 1,399 | 6.6 | 2.63 | <0.0001 | 2.40-2.88 |
| **Previous pneumonia** | 1,561 | 5.1 | 1,572 | 7.4 | 1.31 | <0.0001 | 1.19-1.44 |
| **Smoking history** |  |  |  |  |  |  |  |
| Never | 7,490 | 24.5 | 4,020 | 19.0 | Reference |  |  |
| Ex | 8,781 | 28.8 | 7,491 | 35.4 | 1.05 | 0.138 | 0.98-1.12 |
| Smoker | 6,751 | 22.1 | 4,700 | 22.2 | 1.06 | 0.127 | 0.98-1.14 |
| Unrecorded | 7,510 | 24.6 | 4,944 | 23.4 | 0.24 | <0.0001 | 0.22-0.26 |
| **Socioeconomic status (IMD)** |  |  |  |  |  |  |  |
| 1 (least deprived) | 6,740 | 22.1 | 4,239 | 20.1 | Reference |  |  |
| 2 | 6,695 | 21.9 | 4,513 | 21.4 | 1.05 | 0.211 | 0.97-1.14 |
| 3 | 6,045 | 19.8 | 4,048 | 19.2 | 1.10 | 0.020 | 1.01-1.19 |
| 4 | 5,889 | 19.3 | 4,286 | 20.3 | 1.18 | <0.0001 | 1.09-1.28 |
| 5 | 5,149 | 16.9 | 4,048 | 19.2 | 1.27 | <0.0001 | 1.17-1.38 |
| **Annual GP visits (median, IQR)** | 8 (3-17) | | 11 (5-20) | | 1.02 | <0.0001 | 1.02-1.02 |

- Table S7. Association between ICS and hospitalised pneumonia (Aurum nested case-control)

| **Variable** | **Non-ICS users** | | **ICS users** | | **Adjusted OR** | **p-value** | **95% CI** |
| --- | --- | --- | --- | --- | --- | --- | --- |
|  | **N** | **%** | **N** | **%** |  |  |  |
| **Total** | 43,936 | 54.3 | 36,929 | 45.6 |  |  |  |
| **Mean age (SD)** | 60.3 (19.6) | | 66.1 (17.4) | |  |  |  |
| **Females** | 24,870 | 56.6 | 21,025 | 56.9 |  |  |  |
| **Last ICS** |  |  |  |  |  |  |  |
| No ICS | 43,936 | 100.0 | 0 | 0.0 | Reference |  |  |
| 7-30 days | 0 | 0.0 | 12,533 | 34.2 | 1.81 | <0.0001 | 1.70-1.92 |
| 31-90 days | 0 | 0.0 | 11,056 | 30.2 | 1.63 | <0.0001 | 1.52-1.74 |
| 91-180 days | 0 | 0.0 | 5,928 | 16.2 | 1.24 | <0.0001 | 1.14-1.36 |
| 181-365 days | 0 | 0.0 | 7,087 | 19.4 | 0.93 | 0.116 | 0.85-1.02 |
| **OCS in last year** |  |  |  |  |  |  |  |
| None | 39,579 | 90.1 | 26,438 | 72.2 | Reference |  |  |
| 1 course | 2,340 | 5.3 | 4,956 | 13.5 | 1.72 | <0.0001 | 1.61-1.84 |
| >1 course | 2,017 | 4.6 | 5,210 | 14.2 | 2.33 | <0.0001 | 2.18-2.48 |
| **BMI** |  |  |  |  |  |  |  |
| Normal | 8,909 | 24.1 | 8,909 | 24.1 | Reference |  |  |
| Underweight | 1,026 | 2.8 | 1,026 | 2.8 | 1.69 | <0.0001 | 1.50-1.91 |
| Overweight | 14,616 | 39.6 | 14,616 | 39.6 | 0.79 | <0.0001 | 0.75-0.84 |
| Obese | 10,242 | 27.7 | 10,242 | 27.7 | 0.87 | <0.0001 | 0.81-0.92 |
| Unrecorded | 2,136 | 5.8 | 2,136 | 5.8 | 0.92 | 0.069 | 0.83-1.01 |
| **Diabetes** | 7,366 | 16.8 | 7,836 | 21.4 | 1.14 | <0.0001 | 1.07-1.20 |
| **CVD** | 15,318 | 34.9 | 16,590 | 44.9 | 1.12 | <0.0001 | 1.07-1.18 |
| **CVA** | 1,915 | 4.4 | 2,099 | 5.7 | 1.17 | 0.001 | 1.07-1.28 |
| **Previous pneumonia** | 6,043 | 13.8 | 7,343 | 20.1 | 5.40 | <0.0001 | 5.14-5.66 |
| **Smoking history** |  |  |  |  |  |  |  |
| Never | 15,202 | 34.6 | 9,866 | 26.7 | Reference |  |  |
| Ex | 12,047 | 27.4 | 12,601 | 34.1 | 1.36 | <0.0001 | 1.28-1.44 |
| Smoker | 10,933 | 24.9 | 8,710 | 23.6 | 1.42 | <0.0001 | 1.33-1.51 |
| Unrecorded | 5,754 | 13.1 | 5,752 | 15.6 | 1.11 | 0.009 | 1.03-1.19 |
| **Socioeconomic status (IMD)** |  |  |  |  |  |  |  |
| 1 (least deprived) | 8,689 | 19.8 | 6,616 | 18.0 | Reference |  |  |
| 2 | 8,924 | 20.3 | 7,313 | 19.9 | 1.06 | 0.130 | 0.98-1.14 |
| 3 | 8,608 | 19.6 | 7,025 | 19.1 | 1.12 | 0.004 | 1.04-1.21 |
| 4 | 8,945 | 20.4 | 7,566 | 20.5 | 1.22 | <0.0001 | 1.14-1.32 |
| 5 | 8,746 | 19.9 | 8,324 | 22.6 | 1.36 | <0.0001 | 1.30-1.41 |
| **Annual GP visits (median, IQR)** | 6 (2-14) | | 12 (5-22) | | 1.02 | <0.0001 | 1.02-1.02 |

- Table S8. Association between ICS and PE (Aurum nested case-control)

| **Variable** | **Non-ICS users** | | **ICS users** | | **Adjusted OR** | **p-value** | **95% CI** |
| --- | --- | --- | --- | --- | --- | --- | --- |
|  | **N** | **%** | **N** | **%** |  |  |  |
| **Total** | 8,778 | 45.7 | 10,442 | 54.3 |  |  |  |
| **Mean age (SD)** | 54.6 (18.1) | | 58.3 (16.0) | |  |  |  |
| **Females** | 6,170 | 59.1 | 5,175 | 59.0 |  |  |  |
| **Last ICS** |  |  |  |  |  |  |  |
| No ICS | 10,442 | 100.0 | 0 | 0.0 | Reference |  |  |
| 7-30 days | 0 | 0.0 | 2824 | 32.2 | 1.58 | <0.0001 | 1.40-1.80 |
| 31-90 days | 0 | 0.0 | 2606 | 29.7 | 1.57 | <0.0001 | 1.38-1.78 |
| 91-180 days | 0 | 0.0 | 1392 | 15.9 | 1.45 | <0.0001 | 1.22-1.71 |
| 181-365 days | 0 | 0.0 | 1956 | 22.3 | 0.93 | 0.413 | 0.79-1.10 |
| **OCS in last year** |  |  |  |  |  |  |  |
| None | 9508 | 91.1 | 6342 | 72.3 | Reference |  |  |
| 1 course | 482 | 4.6 | 1236 | 14.1 | 1.53 | <0.0001 | 1.34-1.76 |
| >1 course | 452 | 4.3 | 1200 | 13.7 | 1.91 | <0.0001 | 1.67-2.19 |
| **BMI** |  |  |  |  |  |  |  |
| Normal | 2704 | 25.9 | 1999 | 22.8 | Reference |  |  |
| Underweight | 184 | 1.8 | 195 | 2.2 | 0.86 | 0.443 | 0.60-1.25 |
| Overweight | 4197 | 40.2 | 3538 | 40.3 | 1.30 | <0.0001 | 1.15-1.47 |
| Obese | 2635 | 25.2 | 2579 | 29.4 | 2.04 | <0.0001 | 1.80-2.32 |
| Unrecorded | 722 | 6.9 | 467 | 5.3 | 2.16 | <0.0001 | 1.80-2.60 |
| **CVD** | 4,007 | 38.4 | 4,258 | 48.5 | 0.97 | 0.598 | 0.88-1.08 |
| **CVA** | 477 | 5.4 | 439 | 4.2 | 0.99 | 0.913 | 0.81-1.21 |
| **Previous pneumonia** | 474 | 4.5 | 631 | 7.2 | 1.74 | <0.0001 | 1.49-2.04 |
| **Cancer** | 2,572 | 24.6 | 2,656 | 30.3 | 1.51 | <0.0001 | 1.36-1.68 |
| **Smoking history** |  |  |  |  |  |  |  |
| Never | 3643 | 34.9 | 2449 | 27.9 | Reference |  |  |
| Ex | 2911 | 27.9 | 3014 | 34.3 | 0.98 | 0.768 | 0.88-1.10 |
| Smoker | 2551 | 24.4 | 1995 | 22.7 | 0.87 | 0.022 | 0.76-0.98 |
| Unrecorded | 1337 | 12.8 | 1320 | 15.0 | 1.05 | 0.455 | 0.92-1.21 |
| **Socioeconomic status (IMD)** |  |  |  |  |  |  |  |
| 1 (least deprived) | 2101 | 20.1 | 1657 | 18.9 | Reference |  |  |
| 2 | 2119 | 20.3 | 1689 | 19.3 | 0.83 | 0.013 | 0.72-0.96 |
| 3 | 2034 | 19.5 | 1735 | 19.8 | 0.92 | 0.237 | 0.80-1.06 |
| 4 | 2070 | 19.9 | 1798 | 20.5 | 0.93 | 0.339 | 0.81-1.07 |
| 5 | 2106 | 20.2 | 1889 | 21.5 | 1.04 | 0.588 | 0.91-1.19 |
| **OCP/HRT use** | 460 | 4.4 | 327 | 3.7 | 1.24 | 0.051 | 1.02-1.55 |
| **Annual GP visits (median, IQR)** | 7 (2-15) | | 12 (5-22) | | 1.02 | <0.0001 | 1.02-1.03 |

- Table S9. Association between ICS and CVD (GOLD nested case-control)

| **Variable** | **Non-ICS users** | | **ICS users** | | **Adjusted OR** | **p-value** | **95% CI** |
| --- | --- | --- | --- | --- | --- | --- | --- |
|  | **N** | **%** | **N** | **%** |  |  |  |
| **Total** | 9,449 | 48.10 | 10,196 | 51.90 |  |  |  |
| **Mean age (SD)** | 69.5 (14.0) | | 71.1 (12.8) | |  |  |  |
| **Females** | 4,813 | 50.94 | 5,097 | 49.99 |  |  |  |
| **Last ICS** |  |  |  |  |  |  |  |
| No ICS | 9,449 | 0.00 | 0 | 0.00 | Reference |  |  |
| 7-30 days | 0 | 0.00 | 3,662 | 36.71 | 1.21 | 0.020 | 1.03-1.41 |
| 31-90 days | 0 | 0.00 | 3,285 | 32.93 | 1.29 | 0.001 | 1.11-1.50 |
| 91-180 days | 0 | 0.00 | 1,630 | 16.34 | 0.83 | 0.063 | 0.69-1.01 |
| 181-365 days | 0 | 0.00 | 1,398 | 14.02 | 0.76 | 0.008 | 0.61-0.93 |
| **LABA use** | 0 | 0.00 | 2,308 | 32.08 | 0.81 | 0.023 | 0.68-0.97 |
| **SABA in last year** |  |  |  |  |  |  |  |
| 0-3 | 7,391 | 78.22 | 5,070 | 49.73 | Reference |  |  |
| 4-8 | 1,496 | 15.83 | 3,539 | 34.71 | 0.99 | 0.790 | 0.88-1.10 |
| >8 | 562 | 5.95 | 1,587 | 15.56 | 1.07 | 0.378 | 0.92-1.25 |
| **OCS in last year** |  |  |  |  |  |  |  |
| None | 8,337 | 88.23 | 7,236 | 72.54 | Reference |  |  |
| 1 course | 605 | 6.40 | 1,324 | 13.27 | 1.44 | <0.0001 | 1.24-1.67 |
| >1 course | 507 | 5.37 | 1,415 | 14.19 | 1.33 | <0.0001 | 1.13-1.55 |
| **BMI** |  |  |  |  |  |  |  |
| Normal | 1,704 | 18.03 | 1,635 | 16.04 | Reference |  |  |
| Underweight | 301 | 3.19 | 309 | 3.03 | 0.72 | 0.036 | 0.53-0.98 |
| Overweight | 3,193 | 33.79 | 3,482 | 34.15 | 1.12 | 0.112 | 0.97-1.29 |
| Obese | 2,150 | 22.75 | 2,496 | 24.48 | 1.24 | 0.004 | 1.07-1.44 |
| Unrecorded | 2,101 | 22.24 | 2,274 | 22.30 | 1.32 | <0.0001 | 1.14-1.53 |
| **Diabetes** | 1,461 | 15.46 | 1,607 | 16.11 | 1.04 | 0.561 | 0.92-1.17 |
| **CVD** | 5,439 | 57.56 | 6,038 | 60.53 | 4.61 | <0.0001 | 4.09-5.19 |
| **CVA** | 489 | 5.18 | 559 | 5.60 | 2.61 | <0.0001 | 2.21-3.07 |
| **Smoking history** |  |  |  |  |  |  |  |
| Never | 1,334 | 14.12 | 1,229 | 12.05 | Reference |  |  |
| Ex | 3,939 | 41.69 | 5,036 | 49.39 | 0.91 | 0.188 | 0.79-1.05 |
| Smoker | 2,928 | 30.99 | 2,784 | 27.30 | 1.00 | 0.980 | 0.86-1.16 |
| Unrecorded | 1,248 | 13.21 | 1,147 | 11.25 | 1.18 | 0.061 | 0.99-1.40 |
| **Socioeconomic status (IMD)** |  |  |  |  |  |  |  |
| 1 (least deprived) | 2,120 | 22.45 | 2,167 | 21.73 | Reference |  |  |
| 2 | 2,050 | 21.71 | 2,174 | 21.80 | 1.04 | 0.538 | 0.91-1.19 |
| 3 | 1,930 | 20.44 | 2,093 | 20.99 | 1.03 | 0.660 | 0.90-1.18 |
| 4 | 1,767 | 18.71 | 1,856 | 18.61 | 1.08 | 0.270 | 0.94-1.25 |
| 5 | 1,576 | 16.69 | 1,682 | 16.87 | 1.17 | 0.032 | 1.01-1.35 |
| **Annual GP visits (median, IQR)** | 7 (3-16) | | 12 (5-20) | | 1.03 | <0.0001 | 1.03-1.03 |

- Table S10. Association between ICS and hospitalised pneumonia (GOLD nested case-control)

| **Variable** | **Non-ICS users** | | **ICS users** | | **Adjusted OR** | **p-value** | **95% CI** |
| --- | --- | --- | --- | --- | --- | --- | --- |
|  | **N** | **%** | **N** | **%** |  |  |  |
| **Total** | 3,029 | 50.61 | 2,956 | 49.39 |  |  |  |
| **Mean age (SD)** | 60.3 (19.6) | | 66.1 (17.4) | |  |  |  |
| **Females** | 1,762 | 58.17 | 1,748 | 59.13 |  |  |  |
| **Last ICS** |  |  |  |  |  |  |  |
| No ICS | 3,029 | 0.00 | 0 | 0.00 | Reference |  |  |
| 7-30 days | 0 | 0.00 | 995 | 32.99 | 1.64 | <0.0001 | 1.33-2.03 |
| 31-90 days | 0 | 0.00 | 981 | 32.53 | 1.22 | 0.074 | 0.98-1.53 |
| 91-180 days | 0 | 0.00 | 516 | 17.11 | 0.93 | 0.646 | 0.69-1.26 |
| 181-365 days | 0 | 0.00 | 524 | 17.37 | 0.78 | 0.108 | 0.58-1.06 |
| **OCS in last year** |  |  |  |  |  |  |  |
| None | 2,608 | 88.23 | 2,226 | 73.81 | Reference |  |  |
| 1 course | 176 | 5.95 | 407 | 13.49 | 1.79 | <0.0001 | 1.42-2.26 |
| >1 course | 172 | 5.82 | 383 | 12.70 | 2.62 | <0.0001 | 2.08-3.32 |
| **BMI** |  |  |  |  |  |  |  |
| Normal | 531 | 17.96 | 517 | 17.07 | Reference |  |  |
| Underweight | 64 | 2.17 | 81 | 2.67 | 1.30 | 0.264 | 0.82-2.05 |
| Overweight | 944 | 31.94 | 997 | 32.92 | 0.68 | 0.001 | 0.54-0.86 |
| Obese | 734 | 24.83 | 742 | 24.50 | 0.78 | 0.044 | 0.61-0.99 |
| Unrecorded | 683 | 23.11 | 692 | 22.85 | 1.04 | 0.741 | 0.82-1.32 |
| **Diabetes** | 367 | 12.42 | 467 | 15.48 | 1.08 | 0.488 | 0.87-1.35 |
| **CVD** | 1,268 | 42.90 | 1,557 | 51.62 | 0.99 | 0.892 | 0.81-1.20 |
| **CVA** | 109 | 3.69 | 153 | 5.07 | 1.16 | 0.410 | 0.82-1.64 |
| **Previous pneumonia** | 214 | 7.24 | 214 | 7.10 | 16.03 | <0.0001 | 12.18-21.09 |
| **Smoking history** |  |  |  |  |  |  |  |
| Never | 435 | 14.72 | 427 | 14.10 | Reference |  |  |
| Ex | 1,062 | 35.93 | 1,340 | 44.24 | 1.22 | 0.125 | 0.95-1.59 |
| Smoker | 905 | 30.62 | 822 | 27.14 | 1.50 | 0.002 | 1.15-1.94 |
| Unrecorded | 554 | 18.74 | 440 | 14.53 | 1.03 | 0.846 | 0.76-1.39 |
| **Socioeconomic status (IMD)** |  |  |  |  |  |  |  |
| 1 (least deprived) | 678 | 22.48 | 734 | 24.84 | Reference |  |  |
| 2 | 665 | 22.05 | 653 | 22.10 | 1.13 | 0.308 | 0.89-1.43 |
| 3 | 650 | 21.55 | 639 | 21.62 | 0.95 | 0.653 | 0.75-1.20 |
| 4 | 528 | 17.51 | 500 | 16.92 | 1.54 | <0.0001 | 1.21-1.96 |
| 5 | 495 | 16.41 | 429 | 14.52 | 1.53 | 0.001 | 1.19-1.95 |
| **Annual GP visits (median, IQR)** | 6 (2-15) | | 11 (5-20) | | 1.04 | <0.0001 | 1.03-1.04 |

- Table S11. Association between ICS and CVD (Aurum SCCS)

| **Outcome** | **Time periods** | **Events** | **IRR** | **p-value** | **95% CI** |
| --- | --- | --- | --- | --- | --- |
| **CVD** | 60 days after commencing ICS | 916 | 1.45 | <0.01 | 1.15-1.81 |
|  | Continued ICS |  | 1.27 | <0.01 | 1.08-1.49 |
|  | After stopped ICS |  | 1.08 | 0.55 | 0.85-1.37 |
| **UTI** | 60 days after commencing ICS | 514 | 1.04 | 0.81 | 0.75-1.45 |
|  | Continued ICS |  | 1.03 | 0.82 | 0.83-1.27 |
|  | After stopped ICS |  | 1.47 | 0.01 | 1.09-1.98 |
| **Abdominal pain** | 60 days after commencing ICS | 1790 | 1.07 | 0.41 | 0.90-1.28 |
|  | Continued ICS |  | 1.02 | 0.79 | 0.90-1.14 |
|  | After stopped ICS |  | 0.94 | 0.46 | 0.80-1.10 |
| **Cellulitis** | 60 days after commencing ICS | 209 | 1.48 | 0.10 | 0.92-2.36 |
|  | Continued ICS |  | 1.18 | 0.33 | 0.84-1.66 |
|  | After stopped ICS |  | 1.25 | 0.35 | 0.78-2.02 |

- Table S12. Association between fluticasone compared to beclomethasone, and budesonide compared to beclomethasone (Aurum ICS new-user cohort)

| **Outcome** | **Weighted HR** | **p-value** | **LCI 95%** | **UCI 95%** |
| --- | --- | --- | --- | --- |
| **Budesonide compared to beclomethasone (N=87,630)** | | | |  |
| MACE | 1.24 | 0.466 | 0.69 | 2.23 |
| Arrhythmia | 1.04 | 0.873 | 0.65 | 1.66 |
| PE | 0.49 | 0.110 | 0.20 | 1.17 |
| Pneumonia | 0.94 | 0.799 | 0.60 | 1.48 |
| **Fluticasone compared to beclomethasone**  **(87,639)** | | | |  |
| MACE | 0.85 | 0.488 | 0.54 | 1.34 |
| Arrhythmia | 0.88 | 0.689 | 0.47 | 1.66 |
| PE | 1.09 | 0.780 | 0.59 | 2.02 |
| Pneumonia | 1.31 | 0.130 | 0.92 | 1.85 |
